# Genome misassembly detection using Stash: A data structure based on stochastic tile hashing

**DOI:** 10.1371/journal.pone.0333729

**Published:** 2026-07-13

**Authors:** Armaghan Sarvar, Lauren Coombe, Inanc Birol

**Affiliations:** 1 University of British Columbia, Vancouver, Canada; 2 BC Cancer, Vancouver, Canada; Lucian Blaga University of Sibiu: Universitatea Lucian Blaga din Sibiu, ROMANIA

## Abstract

Analyzing large data from high-throughput sequencing technologies presents significant challenges in terms of memory and computational requirements. It is crucial to develop efficient data structures and computational methods that handle sequencing information. These challenges impact bioinformatics studies, including de novo genome assembly, which serves as a foundation of genomics. Issues like read errors or limitations of heuristic decisions in assembly algorithms lead to genome misassemblies and inaccurate genomic representations, compromising the quality of downstream analyses. Hence, de novo assemblies can benefit from misassembly detection and correction to produce a more optimal assembly. We present Stash, a novel hash-based data structure designed for storing and querying large sequencing data. For an input sequence, Stash uses sliding windows of spaced seed patterns to extract and hash k-mers. The hash values combined with the sequence ID determine the value stored in Stash. A filled Stash can be used to query whether two genomic regions are covered by the same set of reads. This can be used in genome misassembly detection. We demonstrate the effectiveness of Stash in detecting misassemblies in human genome assemblies generated by Flye and Shasta, using Pacbio HiFi reads from the human cell line NA24385. We observed that scaffolding Stash-cut assemblies reduces 7.6% and 3.4% of misassemblies in the Flye and Shasta assemblies, respectively. This is accomplished in 310 minutes utilizing 8 GB of memory. Stash is comparable to alternative long read misassembly correction methods and can result in superior assemblies compared to the baseline.

## Introduction

The rapid advancements in high-throughput sequencing technologies have opened new avenues to be explored using bioinformatics, such as the discovery of complex genomic landscapes or the identification of genetic variations. With associated massive improvements in sequence throughput, storing and analyzing the extracted Gbp-scale genomic information has become a major logistical and computational challenge. Hence, novel data structures and algorithms borrowed from the broader Computer Sciences have been developed to fuel rapid development in addressing these challenges [1]. A particularly computationally demanding challenge is *de novo* genome assembly and the subsequent detection of misassemblies in draft genomes. As these local or long-range errors can impact downstream analyses, detecting them is an important step in assembly validation. Current misassembly detection methods rely heavily on computationally intensive sequence alignments, motivating the need for efficient, alignment-free approaches. Such approaches typically rely on scalable data structures capable of storing and querying large-scale sequencing information, *k*-mer representations (short, length-k substrings of reads) that can tolerate sequencing errors, and the ability to infer regional genomic coverage without explicit alignment.

Consequently, many pipelines use compact indexing structures that prioritize fast *k*-mer queries with low memory overhead. An example is the Bloom filter [2], which is a probabilistic and memory-efficient data structure designed for fast membership queries. Bloom filters can represent large quantities of data succinctly by hashing values and using them as indices in a bit array. In recent years, Bloom filters have been improved to extend their practical utility in scientific research. An example is the multi-index Bloom filter (miBF) data structure [3], which stores a vector of identifiers along with the membership bits, allowing for applications like taxonomy assignment and sequencing read binning without the need for sequence alignment, a costly bioinformatics task in terms of run time and memory. Other advanced data structures used in this field include the quotient filter [4], which utilizes hashing and division operations to store and query data, and the counting quotient filter [5] that also allows efficient counting of the frequency of each element. Cuckoo filters [6] are another improvement over Bloom filters that resolve hash collisions by evicting existing elements from their original locations. There is also a probabilistic indexing data structure called the quasi dictionary [7], designed based on a Minimal Perfect Hash Function, to provide a way to associate any kind of data to a given input key. These data structures enable scalable, alignment-free representations of biological sequence data, making them well suited for large-scale genomic analyses under strict memory constraints.

In practice, the bottleneck in many *k*-mer indexing workflows is the cost of hashing and querying large numbers of overlapping *k*-mers. Many bioinformatics algorithms such as structural variation detection [8,9] or protein function and structure prediction [10,11] are based on identifying *k*-mers. The *k*-mer based methods work by indexing sequence substrings with length *k*, usually in a hash-based data structure. In the querying phase, a given sequence is also segmented into *k*-mers and searched against the index to find shared subsequences. Moreover, hash-based data structures are frequently used to count consecutive *k*-mers for tasks such as similarity searching in large-scale analysis [12]. Accordingly, using high performance hashing algorithms would have a substantial effect on the deployment of these data structures [13]. An example is ntHash2 [14], which is a recursive hashing algorithm based on ntHash [15] tuned for processing DNA/RNA sequences, taking advantage of the k−1 overlap between consecutive *k*-mers. *K*-mer representations therefore serve as a fundamental bridge between biological sequences and scalable, hash-based data structures.

However, *k*-mer–based representations are sensitive to sequencing errors, particularly in settings that rely on noisy long-read sequencing. Hence, some tools, such as the miBF, use spaced seed patterns represented by sequences of zeroes and ones, with ones indicating care and zeroes indicating don’t care positions. Spaced seeds can thus tolerate mismatches, allowing certain base pairs defined by the pattern to mismatch without penalty. This tolerance is particularly important for large-scale analyses of noisy sequencing data, including in *de novo* genome assembly.

*De novo* genome assembly has led to the generation of high-quality reference genomes [16], and it has many applications such as in phylogenetic inference [17], gene annotation [18], and cancer genomics [19]. However, misassemblies remain common due to repeats and other genomic complexities, residual sequencing errors, and assembly pipeline artifacts. Misassemblies can then distort downstream analyses such as inferred structural variation, gene content, and regional coverage patterns. Therefore, detecting and correcting misassemblies is an important step in producing reliable draft genomes. Despite recent improvements in long read sequencing technologies, most practical misassembly detection utilities still rely on aligning reads to the draft assembly, which is computationally expensive for large genomes, motivating scalable alignment-free alternatives.

To discover misassemblies in *de novo* assemblies, some identification methods have been proposed, and they are mostly based on aligning the sequencing reads back onto the assembly to find measurements for assembly quality. Tools such as REAPR [20] or Pilon [21] use short reads to carry out misassembly detection using read to contig alignments. REAPR utilizes a scoring system that assesses both local accuracy and the presence of larger-scale errors within assemblies. In this approach, error-free bases correspond to regions that are likely to be accurate. Conversely, bases failing to meet these criteria receive scores ranging from zero to one, determined by the deviation from acceptable thresholds in metrics such as read depth, characteristics of paired mapping, and the presence of soft clipping. Pilon considers assembly improvements (polishing and error correction) and variant detection as a unified process, starting with an input assembly and employing read alignments to pinpoint discrepancies. In regions with suboptimal alignments, Pilon conducts local reassembly, filling gaps, and capturing large-scale insertions and deletions. This approach encompasses the identification and rectification of base errors and the detection of potential local misassemblies.

Linked reads, a sequencing technology developed by 10x Genomics (Pleasanton, CA, US) for their Chromium platform, can also be used to correct misassemblies, such as in Tigmint [22]. Tigmint identifies drops in molecule coverages inferred from the alignment of linked reads to the assembly. Since linked reads can provide versatile long-range information, they can detect genome misassemblies better than short reads [23].

However, long reads from the third-generation sequencing technologies provide the most comprehensive view of genomic regions. Tools such as Tigmint-long [24], ReMILO [25], Inspector [26], and GAEP [27] have been proposed as long-read misassembly detection tools. Tigmint-long is based on the Tigmint tool explained above and has been adapted to work with long reads. ReMILO is a reference-assisted misassembly detection algorithm that leverages both short reads and PacBio SMRT long reads, utilizing their complementary strengths rather than being exclusive to long-read information. Similar to Tigmint-long, in the Inspector and GAEP tools, sequencing reads are aligned to the assembly. Inspector detects and corrects errors in the assembly by first inferring assembly and read-to-contig alignment statistics such as the read mapping rate and average alignment depth, and then using several rounds of alignment and local assembly to correct the detected misassembly regions. GAEP does not output a corrected assembly, and for reporting detected misassemblies, it leverages the fact that long-read alignments can break at the corresponding misassembly positions. It is worth noting that these tools rely on long-read alignments, which are particularly computationally intensive to generate, especially when working with large genomes. This reliance motivates the exploration of alternative, alignment-free approaches that can scale efficiently under fixed memory budgets.

Here, we introduce stochastic tile hashing (Stashing) to describe large collections of sequences through a lossy and scalable representation. Our proposed novel underlying data structure, Stash, uses a fixed allocated memory, where the memory loci are indicated by some hashed values of subsequences, and the stored value is partly determined by the index of the sequence. The Stash data structure efficiently facilitates saving and retrieving sequence mapping information.

In contrast to membership data structures such as the Bloom filter which let us store and query information regarding the presence or absence of a set of observations, the Stash data structure also provides us with the utility of implicitly storing a property that corresponds to the input observations, such as sequencing reads. When querying the data structure, we can compare the stored property for two observations of interest. For example, Stash can determine if two sequences originate from similar genomic regions without the need for computationally intensive sequence alignment. We illustrate the utility of Stash in detecting and correcting genome misassemblies, focusing on the use of PacBio HiFi sequencing reads. Due to its effectiveness in storing large amounts of regional information, we expect the Stash data structure to have broad applications in other areas of genomics research as well, such as scaffolding and polishing.

## Methods

### Filling the Stash data structure

**Fig 1:**
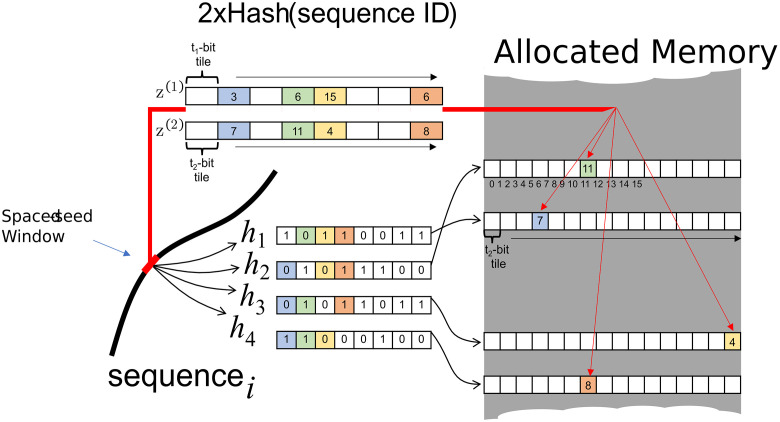
The Stash data structure. Algorithm, data structure, and sequencing-data population process with four spaced seed patterns $\left(h_{\text{1}}-h_{\text{4}}\right)$. The two sequence-ID hashes labeled ${\text{z}}^{\left(\text{1}\right)}$ and ${\text{z}}^{\text{2}}$ (Methods) are partitioned into ${\text{t}}_{\text{1}}$ -bit and ${\text{t}}_{\text{2}}$ -bit tiles used to select the destination column and stored value.

Stash is an R × C two-dimensional bit array of tiles, where each row contains $C=\;=\;{\text{2}}^{{\text{t}}_{\text{1}}}$ tiles (columns) addressed by ${\text{t}}_{\text{1}}$ bits, and each tile stores ${\text{t}}_{\text{2}}$ bits. To populate Stash for a sequence with identifier *ID*, we (i) hash *ID* with two independent hash functions and partition the resulting hash values into tile arrays that provide the destination column and the value to write, and (ii) scan the sequence with sliding windows under *h* symmetric spaced-seed patterns, using a canonical ntHash2 value per window to select the row(s) updated. At each sliding-window position, each spaced-seed pattern updates one row, and the set of these *h* accessed rows is referred to as a *Stash frame*. Using multiple spaced-seed patterns reduces the probability that distinct *k*-mers map to the same Stash frame due to ntHash collisions. The remainder of this section formalizes the deterministic mapping from $\left(ID,\;{\text{x}}_{\text{i}}(\text{p})\right)$ to a tile update (Fig 1).

**Fig 2:**
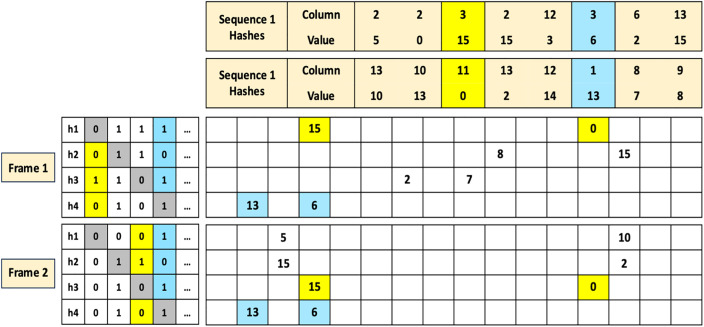
The results of sample frames after filling Stash with two overlapping sequencing reads. The colors show equal hash outputs resulting in the same columns and tile values extracted from the sequence ID for the overlapping region.

For each spaced seed pattern, to determine the destination tile and its content to be stored in the selected Stash frame row, the hashes corresponding to the other spaced seed patterns are combined with the two sequence ID hash values (as defined below). More specifically, for the *i*th spaced seed pattern and its corresponding hash value, the designated most significant bits of the other hash values are concatenated to create an index addressing the tiles in the two sequence ID hashes. One addressed tile specifies the column, while the other indicates the value to be stored in a Stash tile. To formalize this ma*p*ping, let $g_{\text{1}}\;$ and $g_{\text{2}}$ be two independent hash functions applied to the sequence identifier ID. Define ${\text{z}}^{\left(\text{1}\right)}=\;g_{\text{1}}(ID)$ and ${\text{z}}^{\left(\text{2}\right)}=\;g_{\text{2}}(ID)$, where each is a fixed-width T-bit hash value. Partition ${\text{z}}^{\left(\text{1}\right)}$ into consecutive non-overlapping $t_{\text{1}}$ -bit tiles U[0],U[1],… and partition ${\text{z}}^{\left(\text{2}\right)}$ into consecutive non-overlapping $t_{\text{2}}$ -bit tiles V[0],V[1],…, using the same tile order (*M*SB-first) for both; let $M=\text{ min}(\lfloor \frac{\text{T}}{{\text{t}}_{\text{1}}}\rfloor ,\;\lfloor \frac{\text{T}}{{\text{t}}_{\text{2}}}\rfloor )\;$ denote the number of tile indices available to both partitions. For a sliding window position *p* and spaced-seed pattern $h_{i}$ (as in Fig 1), let *x*_*i*_ (*p*) denote the canonical ntHash2 output under $h_{i}$ (canonicalized by selecting a single representative hash per window as described above), and map it to a row index $r_{i}(p)=\;x_{i}(p)\;mod\;R.$ Next, construct a tile selector by taking bit position *i* from each other seed hash $x_{j}(p)$ for j ≠ i (where bit positions are MSB-indexed with position 1 denoting the most significant bit), concatenating these (h−1) bits in the fixed order (1,…,i−1,i+1,…,h), interpreting the result as an integer, and reducing it modulo *M* to obtain $j_{i}(p)$, where *h* is the number of spaced-seed patterns. The destination column is $c_{i}(p)=U\left[j_{i}(p)\right]\;mod\;C\;$ and the value written to that location is $v_{i}(p)=\;V\left[j_{i}(p)\right]$, yielding a fully specified mapping from $\left(x_{i}(p),\;ID\right)$ to a deterministic $\left(r_{i}(p),\;c_{i}(p),\;v_{i}(p)\right)$ tile update. As an example of how Stash is filled, we populate the data structure with two sequencing reads and show how it leads to repeated patterns when the two spaced seed sliding windows fall in an underlying region where the sequences overlap (Fig 2).

### Querying the Stash data structure

To query the Stash data structure and answer the question of whether two genomic regions are potentially covered by the same set of sequencing reads, we define a score metric called the “number of matches”. This metric captures the similarity between any two populated Stash frames, or between two windows composed of frames.

#### The number of matches for two frames.

Based on the Stash filling process, for any two spaced seed pattern outputs coming from the same input sequence, we expect the two corresponding Stash frames to contain a number of equal tile values when examining the two frame columns extracted with the same index. More specifically, it is likely to observe equal pairs of column and tile values when comparing their corresponding Stash frames, as they access the same sequence ID hash vectors when being inserted into Stash.

Hence, the number of matches similarity metric for a pair of frames is calculated by counting the tiles with equal values in each Stash frame column. Specifically, the number of matches between two frames is incremented if a tile in one frame has the same value as at least one tile in the corresponding column of the second frame. This ensures the commutative property when counting the number of matches between two Stash frames.

To formalize the probabilistic behavior of the number of matches metric, we model the match count M between two frames under a null hypothesis that the underlying sequences are unrelated (i.e., not covered by overlapping reads from the same genomic region).

***Null model (unrelated sequences):*** Consider two frames *A* and *B*, each consisting of *h* rows and *C* columns. We define *T* as the number of tile positions (i,j)  in frame A (out of *hC* total) for which the tile value ${\text{A}}_{\text{ij}}\;$ matches at least one of the *h* tile values in column *j* of frame *B.* If the sequences are unrelated, they originate from reads with different sequence IDs, and thus the tile values stored at each position are determined by independent sequence ID hashes. Assuming tile values are uniformly distributed over ${\text{2}}^{{\text{t}}_{\text{2}}}\;$ possible values, the probability that a specific tile from frame *A* matches a specific tile in the same column of frame *B* is:


$$\begin{array}{c} \;\pi \;=\;{\text{2}}^{-t_{\text{2}}} \end{array}$$ (1)

For a single column containing *h* tiles in each frame, the probability that a specific tile from frame *A* matches at least one of the *h* tiles in the same column of frame *B* is:


$$\begin{array}{c} q\;=\;\text{1}\;-\;(\text{1}\;-\;\pi {)}^{h} \end{array}$$ (2)

Under the null hypothesis, the number of matching tiles *T* across all *hC* tile positions can be approximated by a binomial distribution:


$$\begin{array}{c} M\;\sim \;Binomial(hC,\;q) \end{array}$$ (3)

with expected value E[M] = hC · q   and variance Var[M] = hC · q· (1 − q).

***Alternative model (related sequences):*** When two frames originate from *k*-mers covered by overlapping reads sharing sequence IDs, the tile values are determined by the same ID hash vectors. In the ideal case, tiles at corresponding positions would be identical, yielding *T* ≈ *hC*. In practice, hash collisions and Stash saturation (Equation 4) reduce the observed match count, but *T* typically remains substantially above the null expectation.

**Fig 3:**
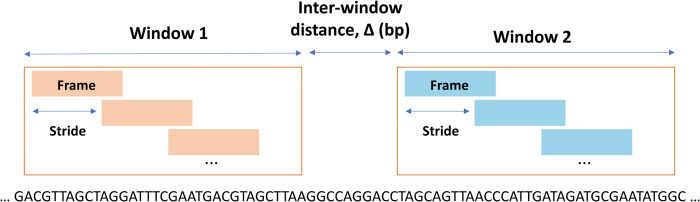
A pair of windows of Stash frames.

The utility of the number of matches metric derives from the separation between the null distribution (unrelated sequences, T ~ Binomial(hC, q)) and the alternative distribution (related sequences, with substantially elevated M). Fig 3 demonstrates this separation by showing match count distributions at various inter-window distances Δ, which converge to the empirically estimated unrelated-window distribution as Δ → ∞. The null model provides a theoretical baseline: match counts near E[*M*] suggest unrelated sequences, while substantially higher counts indicate shared read coverage. For misassembly detection, we threshold the match signal to identify positions where counts drop below a specified cut-off. The threshold balances sensitivity to detect true misassemblies against specificity to avoid excessive fragmentation and is determined empirically through parameter sweeps optimizing contiguity versus accuracy trade-offs. Supplementary Method 2 in S1 File provides the mathematical framework for threshold selection based on the null distribution.

#### The number of matches for windows of frames.

To have a more general view of a genomic region, we define windows of Stash frames. A window consists of a specific *number of frames* each starting at a distance called the *stride* from the starting point of the previous frame. Any two sliding windows of frames have an adjustable distance of *delta*. Fig 3 shows a diagram of two windows placed over a sequence.

For two windows of frames, we calculate the aforementioned number of matches score for every pair of frames residing in the windows and take the maximum score among them, which indicates how likely it is that the underlying sequences of the two windows originate from the same genomic region. When comparing two windows each containing f frames, we compute $f^{2}$ pairwise match counts between all frame pairs. Under the null hypothesis (unrelated windows), each pairwise match count can be approximated by the frame-level null model T  ~ Binomial(hC, q);  however, these scores are not strictly independent because frame pairs can share rows and are affected by saturation and overwrites. Taking the maximum across $f^{2}$ pairs increases the expected null maximum, but improves sensitivity when any frame pair reflects shared read coverage, yielding stronger discrimination than single-frame comparisons. Supplementary Fig 1 in S1 File demonstrates the effectiveness of this metric.

### Stash properties

#### Saturation.

The Stash data structure can get saturated if the same *k*-mers are repeatedly added. This is particularly relevant when the sequence coverage depth is high in a given read set. We define saturation as the probability of a row being filled completely by the resulting hashed values of spaced seed pattern outputs. Assuming Stash has *C* columns, the probability of inserting into a row tile would be 1/C . If we define the number of insertions of the same spaced seed as i, the Stash row occupancy would be based on the following formula (Equation 4).


$$\begin{array}{c} Stash\;Row\;Occupancy=\text{1}-(\text{1}-\frac{\text{1}}{C}{)}^{i} \end{array}$$ (4)

#### Time complexity.

For an input sequencing read set of average length $l_{avg}$ and $n_{r}$ reads, assuming ntHash2 is used, the time complexity of filling the data structure would be as follows.


$$\begin{array}{c} T_{f}=O(h^{\text{2}}l_{avg}n_{r}) \end{array}$$ (5)

where *h* is the number of spaced seed patterns applied to the sequence before using ntHash2, i.e., the number of frame rows. Here, we can see that the time complexity of filling Stash is linear with respect to the size of the input data.

On the other hand, considering two input *k*-mers and their corresponding extracted frames, the time complexity of comparing the two frames by hashing the sequences and counting the number of matches would be


$$\begin{array}{c} T_{cf}=O\left(h^{\text{2}}C\right)+O(hk) \end{array}$$ (6)

Additionally, when comparing two windows, i.e., sets of frames, as all pairs of frames will be examined at a time, by defining the *number of frames* in each window as f, and assuming that h×C is larger than k, the time complexity would be


$$\begin{array}{c} T_{cw}=O\left({f^{\text{2}}h}^{\text{2}}C\right) \end{array}$$ (7)

### Genome misassembly detection and correction

After populating the Stash data structure with a set of sequencing reads based on the steps explained in Section 2.1, and given the corresponding input genome assembly for the reads, we extract the matches signal of each assembly contig by sliding a pair of Stash windows across the contig and computing the number of matches metric for each position. Next, we apply max pooling over the extracted signal to reduce noise and disregard unexpectedly low number of matches values, which arise due to genome repeats and complexities or assembly method artifacts. More specifically, a window is defined with the size of the pooling region, which specifies the number of consecutive base pairs that will be compared together. It is then slid over the signal, extracting the maximum value within each region, and this process is repeated until the entire signal is covered.

Next, we compare each value of the generated signal with a threshold specified based on the expected number of matches between two unrelated windows of frames. If the value is less than the defined threshold, Stash reports a misassembly position since the two inspected windows of frames are likely not covered by the same set of sequencing reads and the underlying sequences do not originate from neighboring genomic regions. As a result, Stash cuts the assembly at the detected positions with low number of matches values using a module called StashCut. Breaking misassemblies allows the resulting sequences to be correctly joined during subsequent scaffolding.

### Experiments

Our experiments show how Stash is able to probabilistically infer whether two genomic regions originate from the same set of sequencing reads. For demonstration, we consider a Stash with ${\text{2}}^{\text{30}}$ rows and 16 four-bit tiles and fill it with ~30-fold coverage Pacbio HiFi long sequencing reads from the human cell line NA24385 [28] (Supplementary Table 1 in S1 File). The Stash dimensions were chosen in order for the Stash frames to be saturated based on the coverage of the input read set (refer to Equation 1), while also limiting the total memory usage of Stash to 8 GB, a value small enough to fit on many consumer-level computers. We use four symmetrical spaced seeds of length 26 and weight 18 to hash each sequence *k*-mer. The number of spaced seeds used balances ensuring robustness to mismatches without dedicating too much memory for any single Stash frame.

For the first experiment, we define a set of window distances (*delta*). Then, for each distance, we randomly choose 10,000 positions on the read set, a quantity that we empirically found large enough to ensure a robust representation of the distribution. This results in a match distribution for each *delta*. We also prepare a 10,000-point distribution for completely unrelated windows which can be conceptualized as a *delta* of *infinity*. This was simulated by randomly placing the two windows over reads of different chromosomes.

To showcase the performance of Stash in misassembly detection, we analyze human genomes assembled by Flye [29] and Shasta [30] using the same read set used to filled the Stash. Both assemblies are also scaffolded using ntLink [31], a recent long-read genome scaffolder, to give us a total of four baseline assemblies (refer to Supplementary Table 2 in S1 File for details of each assembly). On both Flye and Shasta assemblies, we explored ${\text{5}}^{\text{5}}=\text{3},\text{125}$ parameter configurations of StashCut, representing a coarse grid search over five parameters each with five possible values, as described in Supplementary Method 1 in S1 File. We then perform ntLink scaffolding on each run and use QUAST [32] to evaluate the final assemblies, utilizing the reference genome GRCh38 to generate statistics such as contiguity metrics and the number of misassemblies.

Finally, a default parameter set for StashCut is chosen as discussed in the Results section and used to compare the performance of Stash-based misassembly correction with Tigmint-long and Inspector.

All benchmarking tests have been performed on a server-class system with 128 Intel(R) Xeon(R) CPU E7-8867 v3 @ 2.50 GHz with 2.4 TB RAM.

## Results

### Match signal and threshold calibration

**Fig 4:**
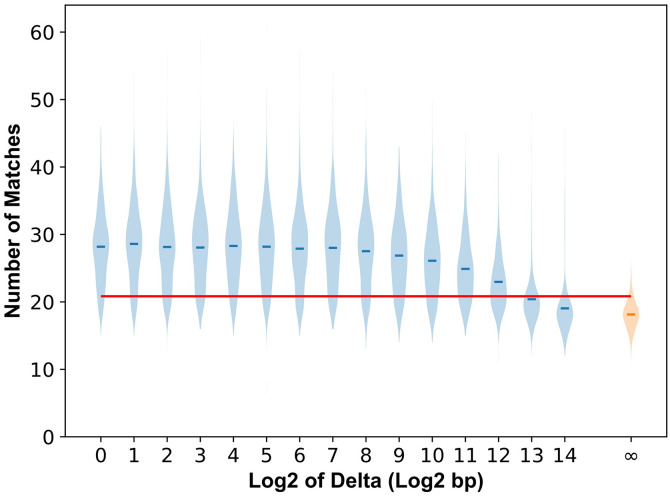
The distribution of the number of matches between two windows of frames as we increase the distance between them. Distance of infinity represents windows from sequencing reads of different chromosomes.

As Δ increases, the match distributions between window pairs converge toward the distribution for unrelated windows (Δ = ∞), indicating the windows are more likely from non-adjacent genomic regions; operationally, we mark such cases using the 90% prediction-interval threshold derived from the unrelated-window distribution (Fig 4). This convergence is consistent with our null model (Methods), where unrelated sequences produce match counts following a binomial distribution. The empirical distribution at Δ = ∞ closely approximates the theoretical null expectation. This experiment demonstrates the capability of the Stash data structure to distinguish between related and unrelated windows within a queried sequence. As detailed in Supplementary Table 4 in S1 File, for delta values less than ${\text{2}}^{\text{11}}=\text{2},\text{048}$, between 81.7% and 91.8% of all matches are already higher than the 90% prediction interval threshold (the drawn red line). Supplementary Table 5 in S1 File shows for delta values less than 512, between 70.5% and 79.7% of all matches are higher than the 99% prediction interval threshold.

### Parameter space exploration identifies high-performing regimes

**Fig 5:**
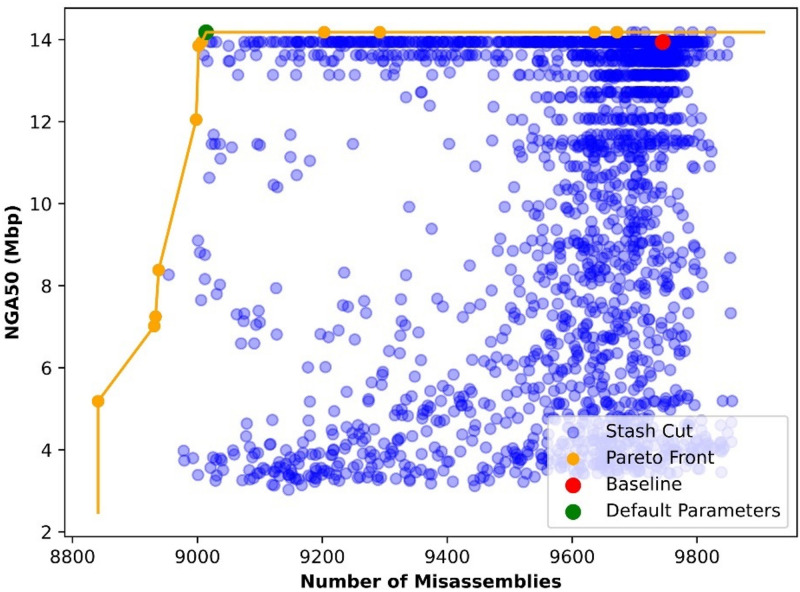
StashCut applied with 3,125 different configurations on the Flye assembly. Results were obtained using QUAST after scaffolding the assemblies using ntLink. As displayed by the Pareto frontier, higher quality cuts result in lower number of misassemblies and higher NGA50 length.

The StashCut misassembly detection is controlled by five parameters, namely *number of frames*, *stride*, *delta*, *threshold*, and *kernel radius*. Fig 5 shows that many explored configurations of StashCut from the parameter space of

Table 1 will either overcut the Flye assembly (the bottom left region) and therefore have low misassemblies and low contiguity represented by NGA50, or undercut and leave both metrics high (the top right region). There are, however, configurations that are superior to the others, considerably reducing extensive misassemblies without sacrificing the contiguity.

**Table 1:** The empirically chosen parameter space for StashCut. The underlined values indicate values that were never seen on the pareto front, and the bolded numbers show the chosen default StashCut parameter set (refer to Fig 5).

Parameter	Explored Values
Number of Frames	2, **4**, 6, 8, 10
Stride (bp)	1, 20, **40**, 60, 80
Delta (bp)	1, 10, **100**, 1000, 10000
Threshold	17, 18, 19, **20**, 21
Kernel Radius (bp)	1, **2**, 3, 4, 5

These configurations are located at the top left region of Fig 5. The default parameter set for Stash corresponds to the optimized configuration chosen from the Pareto frontier, as shown in Table 1. Supplementary Fig 2 in S1 File displays the same parameter sweep results on the Shasta assembly.

**Fig 6:**
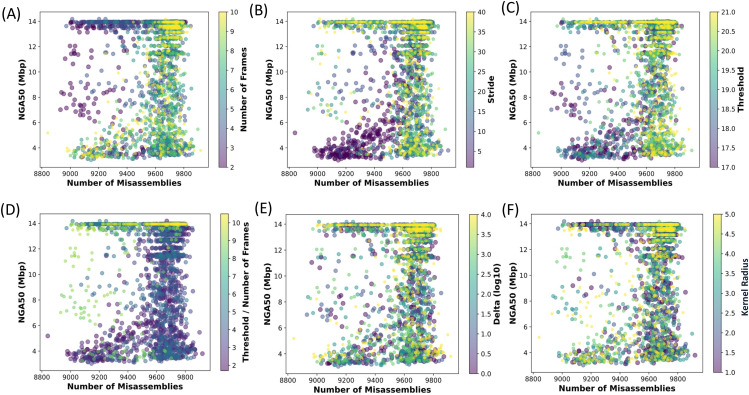
The effect of each Stash parameter on the scaffolded result after StashCut. Some parameters are easy to justify, while others do not seem to have an immediately obvious pattern. Panel (D) represent both the threshold and the number of frames parameter. For improved visibility, points with higher color values are shown in front while points with lower color values have larger sizes.

To understand the effect of each Stash parameter, we color-coded the data points from Fig 5 and show them in Fig 6. In this figure, a higher density of the configurations is on the top left region, and brighter points are shown on top of the darker points. As suggested in Fig 6A, the *number of frames* parameter should be kept under five to achieve the highest performance. This is sensible as a high *number of frames* will cause the window maximum operator to fade out the details of the matches signal. Moreover, Fig 6B indicates that low *stride* values (<~20) are not effective, suggesting that a window that spans over a larger genomic region might be more resistant to sequencing errors. For example, if a frame of a window contains an indel error, with a low *stride*, the following frames of the same window will also include that error, thereby, resulting in a Stash cut in the region, reducing the contiguity without cutting at a true misassembly. Fig 6C suggests higher cut thresholds, which would result in higher contiguity as a result of lower number of false positive cuts. It is worth mentioning that the *threshold* parameter is dependent on the *number of frames* parameter. A good *threshold* value is defined based on the expected number of matches between a pair of unrelated windows. Fig 6D combines *threshold* and *number of frames* to show this dependency, and indicates that high thresholds paired with low *number of frames* and low thresholds paired with high *number of frames* will not result in the optimum cut. Supplementary Figs 3 and 4 in S1 File further demonstrate the optimal *threshold* value through precision-recall curves which are generated using custom definitions for precision and recall (Supplementary Method 1 in S1 File). Fig 6E does not show an obvious pattern for the *delta* parameter. The only consideration for this parameter is that the value needs to be set with regards to the minimum contig length. As shown in Fig 6F, higher *kernel* radius values result in the same generalization and loss of details as high *number of frames* values.

### Benchmarking, breakpoint concordance, and generalizability

**Fig 7:**
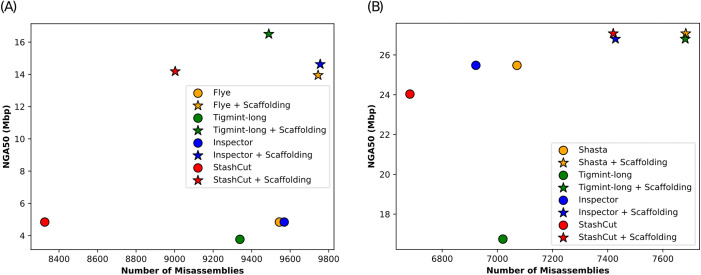
Performance comparison between StashCut and other misassembly correction methods on Flye (A) and Shasta (B) assemblies. Each figure contains two set of evaluations; circles represent the corrected assembly while stars include an additional scaffolding step after correction. StashCut, represented in red, achieves the least number of misassemblies both before and after scaffolding, and Tigmint-long, represented in green, achieves the most contiguous scaffolded Flye assembly. On the Shasta assembly, StashCut results in an objectively better scaffolded assembly than the baseline shown in orange.

Using the default parameter set identified from this parameter exploration (Table 1), we next compared StashCut’s performance against established alignment-based misassembly correction tools. As can be seen from Fig 7, StashCut produces quantifiable trade-offs relative to both Tigmint-long and Inspector. For the Flye assembly of human cell line NA24385 data, StashCut reduces extensive misassemblies by 13% while maintaining the NGA50 length metric (RR 0.872, 95% CI [0.847, 0.898], p = $\text{8}.\text{9}\text{0}\times {\text{1}\text{0}}^{-\text{2}\text{0}};\;$Supplementary Tables 6 and 17 in S1 File). On the same dataset, Inspector results in < 0.3% change to both NGA50 and the number of misassemblies, and Tigmint-long results in a 2% reduction in misassemblies with the downside of reducing the NGA50 by 22%. By performing ntLink scaffolding on the baseline Flye assembly and the output of each misassembly correction module, we can see that the scaffolded StashCut assembly still has 7.6% fewer misassemblies compared to the baseline scaffolded Flye (9,003 vs. 9,745) (RR 0.924, 95% CI [0.898, 0.951], p = $\text{6}.\text{6}\text{0}\times {\text{1}\text{0}}^{-\text{8}}$; Supplementary Tables 7 and 17 in S1 File), exceeding the corresponding changes for Inspector (−0.1%) and Tigmint-long (2.6% reduction) (Supplementary Table 7 in S1 File). However, this improvement comes at the cost of lower NGA50 compared to the other methods (StashCut: 14.2 Mbp, Inspector: 14.6 Mbp, Tigmint-long: 16.5 Mbp) as shown in Supplementary Table 6 in S1 File. When correcting the Shasta assembly, StashCut reduces extensive misassemblies from 7,071 (baseline Shasta) to 6,683 (−388, 5.5% reduction) (Poisson rate ratio (RR) of extensive misassemblies per Gbp assembled, relative to baseline: 0.945; 95% CI [0.914, 0.977], p = $\text{9}.\text{6}\text{7}\times {\text{1}\text{0}}^{-\text{4}}$; Supplementary Tables 8 and 17 in S1 File), compared to Inspector (6,922, −149, 2.1% reduction) and Tigmint-long (7,020, −51, 0.7% reduction) (Supplementary Table 8 in S1 File). This reduction is accompanied by an NGA50 decrease from 25.5 Mbp (baseline) to 24.0 Mbp for Shasta + StashCut (−5.7%), whereas Inspector maintains NGA50 at 25.4 Mbp and Tigmint-long decreases NGA50 to 16.7 Mbp (Supplementary Table 8 in S1 File). After ntLink scaffolding, Shasta + ntLink has 7,683 extensive misassemblies, and Shasta + StashCut + ntLink reduces this to 7,420 (−263, 3.4% reduction) (RR 0.966, 95% CI [0.936, 0.997], p = 0.033; Supplementary Tables 9 and 17 in S1 File), which is comparable to Shasta + Inspector + ntLink (7,428, −255, 3.3% reduction) and larger than Shasta + Tigmint-long + ntLink (7,681, −2, 0.03% reduction) (Supplementary Table 9 in S1 File). In this scaffolded setting, the NGA50 is 27.1 Mbp for both Shasta + ntLink and Shasta + StashCut + ntLink, while Inspector and Tigmint-long yield NGA50 values of 26.8 Mbp and 26.8 Mbp, respectively (Supplementary Table 9 in S1 File).

To compare the misassemblies identified by StashCut and the alignment-based methods, we evaluated breakpoint coordinates using a ± 1 kb overlap criterion on Flye and Shasta assemblies (Supplementary Results 4 in S1 File). On Flye, 61.2% of StashCut breakpoints overlapped Inspector and 64.9% overlapped Tigmint-long (Supplementary Table 15 in S1 File), while StashCut additionally reported 3,226 (38.8%) and 2,926 (35.1%) of StashCut breakpoints not detected by Inspector and Tigmint-long, respectively. Inspector and Tigmint-long showed higher concordance with each other (75.2%, Jaccard = 0.62), whereas agreement between StashCut and alignment-based methods was lower (Jaccard = 0.40–0.44). On Shasta, the overlap increased to 68.1% with Inspector and 70.0% with Tigmint-long (Supplementary Table 16 in S1 File), but StashCut still identified 2,133 (31.9%) and 2,003 (30.0%) unique breakpoints not found by Inspector and Tigmint-long. Alignment-based methods again showed higher mutual overlap (78.7%, Jaccard = 0.66), matching the pattern seen with the Flye assemblies.

To complement our results, we also evaluated StashCut’s performance using Oxford Nanopore Q20 + reads from the publicly available HG002 dataset, released by Oxford Nanopore Technologies. While ONT reads generally exhibit higher error rates—especially indels in homopolymer regions—they offer ultra-long read lengths and continue to gain popularity in *de novo* assembly workflows. On the ONT Q20 + HG002 Flye baseline assembly, StashCut reduced QUAST extensive misassemblies from 918 to 814 (−11.3%) while preserving contiguity (baseline NG50 ~ 5.10 Mb; NGA50 ~ 4.65 Mb; StashCut NG50 ~ 5.02 Mb, NGA50 ~ 4.65 Mb) (Supplementary Results 1 in S1 File).

In addition to human genome datasets, we further evaluated StashCut’s performance on a non-human model organism. Using ~428-fold coverage PacBio RSII reads from *C. elegans* (SRR7594465), we applied StashCut to an existing assembly (ASM1813679v1) and assessed its performance using the WBcel235 reference. This experiment demonstrates StashCut’s applicability beyond human genomes. StashCut reduced QUAST extensive misassemblies in ASM1813679v1 from 242 to 231 (−4.5%) while maintaining an NG50 of 393,294 bp and yielding an NGA50 of 334,673 bp, and after ntLink scaffolding reduced extensive misassemblies from 245 to 231 (−5.7%) (Supplementary Results 2 in S1 File).

To further assess the generalizability of our tool, we performed two supplementary evaluations, targeting robustness across genome scale and sequence context. First, we evaluated StashCut on the human mitochondrial genome by extracting chrM reads from NA24385 after mapping to GRCh38 (Supplementary Results 5 and Supplementary Table 18 in S1 File). With window parameters scaled for the 16.6 kb genome, StashCut preserved the Flye mtDNA assembly without fragmentation; because the baseline mtDNA assembly contained no extensive misassemblies, this analysis primarily tests that StashCut does not introduce spurious breaks in a small-genome setting and therefore serves as a robustness check. Second, we intersected QUAST extensive misassembly breakpoints with GIAB difficult regions (tandem repeats, homopolymers, segmental duplications, and low-mappability; Supplementary Results 6 and Supplementary Table 19 in S1 File) to assess whether there was any bias toward, or against, corrections at these loci. Among 1,218 extensive misassemblies corrected by StashCut in the Flye assembly, 701 (57.6%) overlapped difficult regions, similar to baseline (60.9%; 5,815/9,544), indicating no strong bias toward or against repetitive/structurally complex contexts.

To assess the performance of StashCut with lower coverage input reads, we downsampled the ~ 30 × long-read dataset used for StashFill to ~15× and ~7.5 × coverage (50% and 25% of reads), while holding the input assembly fixed (Supplementary Results 3 and Supplementary Table 14 in S1 File). Misassembly reduction attenuated with decreasing depth

but remained significant (RR 0.872 at ~30 × , 0.922 at ~15 × , 0.959 at ~7.5×), while NGA50 remained ~4.8 Mbp across the tests, indicating no evidence of fragmentation.

Filling the Stash with the sequencing data used in our experiments takes 291:34 minutes using 8 threads and consumes a peak memory of 8.02 GB. Table 2 shows the benchmarking results for all three misassembly correction modules on the Flye assembly using 8 threads. The default configuration of StashCut, with number of frames equal to 4 takes 18:42 and consumes 8.74 GB peak memory. Considering an end-to-end workflow that includes StashFill + StashCut, the total time is 310:16, which is 6.5-fold longer than Tigmint-long (47:41) while using about 47% less peak memory (8.74 GB vs. 16.58 GB). Relative to Inspector, the same end-to-end workflow is 1.5-fold faster (310:16 vs. 458:42) and uses about 70% less peak memory (8.74 GB vs. 29.53 GB). If the filled Stash is reused across multiple correction runs (in parameter sweeps or multiple assemblies from the same read set), the one-time StashFill cost is amortized. The table also includes the benchmarking results for a StashCut with number of frames equal to 1 (1:57, 8.72 GB) in order to show that increasing frames from 1 to 4 increases runtime from 1:57–18:42 (Table 2), consistent with the expected superlinear dependence on the *number of frames* parameter. For completeness, model-based confidence intervals and rate-ratio tests for the QUAST extensive misassembly comparisons (Tables 6–9) are summarized in Supplementary Table 14 in S1 File.

**Table 2:** Benchmarking results for Stash, Inspector, and Tigmint-long applied on the Flye assembly using 8 threads. Note that the complete Stash pipeline requires both StashFill and StashCut.

Method	Time (MM:SS)	Peak Memory (GB)
StashFill	291:34	8.02
StashCut ($n_{f}=\text{1}$)	1:57	8.72
StashCut ($n_{f}=\text{4}$)	18:42	8.74
Tigmint-long	47:41	16.58
Inspector	458:42	29.53

## Discussion

The ability to implicitly store an arbitrary property (such as coverage) from a large set of inputs (like a sequencing read set) is a primary contribution of Stash, making it a useful tool for bioinformatics applications, especially for the genome assembly pipeline. We show how a StashCut-corrected assembly leads to an improved post-scaffolding result due to a reduction in the number of misassemblies. Compared to the other state-of-the-art misassembly detection methods such as Tigmint-long or Inspector, Stash does not rely on any sequence alignment; moreover, it has a fixed, user-parameterized memory footprint and a runtime that scales with the size of the input read set. This makes resource use predictable and enables direct, quantitative comparison of runtime and memory against alignment-based correction tools.

Stash presents another notable advantage which is not being confined to a specific input read length category. We anticipate its efficacy across short reads, linked reads, and long reads. For short reads, the same filling and querying approach can be used, and for linked reads, the associated read barcodes can be hashed and stored instead of read IDs. Our findings in this study demonstrate the robust performance of Stash within long-read assembly pipelines due to their long-range information. This is important as the continuing progress in accuracy, throughput, and cost reduction have made long-read sequencing useful in genomics.

The quality of the reads has a direct effect on the performance of Stash, as the match-count signal can be vulnerable to sequencing errors. Sequencing errors reduce concordance among overlapping reads, which can manifest as lower and more variable numbers of matches in regions with indels, gaps, and mismatches. In our experiments, we primarily used ~30-fold coverage PacBio HiFi long-read sequencing data from the human cell line NA24385, with an average Phred quality score of 35, consistent with high-accuracy long-read sequencing [33,34]. PacBio HiFi reads are particularly well suited for misassembly detection due to their low error rates. Additionally, the uniform coverage of these input reads is sufficient to yield high occupancy of Stash frames, considering the allocated frame dimensions in memory. Due to this sensitivity to sequencing errors, we expect the number of matches signal extracted from Stash to have lower number of false positive drops when the data structure has been filled with high-quality reads.

In the conducted experiments, Stash reduces the number of misassemblies in an input assembly of interest without noticeably decreasing the contiguity measured by the NGA50 or NG50 metrics. In our benchmarks, we filled the data structure with the input sequencing reads and applied the StashCut module to the corresponding long read assemblies generated by the Flye and Shasta assemblers. By breaking misassemblies, the resulting subsequences can potentially be correctly joined during subsequent scaffolding. We passed the post-StashCut assembled genome to ntLink scaffolding and used the output to assess the quality of StashCut in the *de novo* assembly pipeline.

To address lower-quality read regimes and varying coverage explicitly, we evaluated StashCut beyond the ~ 30 × PacBio HiFi NA24385 setting used in the main benchmarks. First, we report results on higher-error Oxford Nanopore long reads (HG002) and on a non-human genome using PacBio RSII reads for C. elegans (Supplementary Results 1–2 in S1 File), demonstrating applicability across platforms and sample types. Second, we quantify performance under reduced coverage by downsampling the read set used for StashFill while holding the input assembly fixed (Supplementary Results 3 and Supplementary Table 14 in S1 File). Correction efficacy decreases with depth (RR 0.872 at ~30 × , 0.922 at ~15 × , 0.959 at ~7.5 × ; 1,218 vs 394 corrected misassemblies at ~30× and ~7.5×), while NGA50 remains ~4.8 Mbp, indicating no evidence of fragmentation. Together, these analyses reflect expected operating behavior: as error rates increase or coverage decreases, separation between related and unrelated match-count distributions (Fig 4) shrinks, reducing sensitivity at fixed parameters and motivating calibration of thresholds from the unrelated distribution of the target read set.

Regarding future work, several extensions are worth exploring. First, additional signal-processing methods could be applied to the number-of-matches signal to improve breakpoint localization and reduce sensitivity to localized noise. In particular, region-specific threshold calibration using locally estimated null distributions (for example, per chromosome or stratified by coverage depth and GC content) could help distinguish true misassemblies from drops driven by local sequencing properties. Second, machine-learning approaches could be applied to Stash fingerprints to learn patterns associated with misassemblies and improve detection accuracy. Third, because Stash is a probabilistic data structure, ensemble strategies that combine multiple independent Stash instances may further improve robustness in some settings. Finally, it will be valuable to evaluate Stash in additional genomics applications, including scaffolding, phasing, and read binning for metagenome assembly.

Stash is a novel hash-based data structure used for storing and retrieving large amounts of sequence mapping information such as genome sequencing reads using a lossy representation, allowing for the implicit storage of a specific property associated with the input observations. The successful utility of Stash in the misassembly detection problem has been explored when the stored property is *coverage by a read set*.

## Supporting information

S1 File Supplementary information for “Genome misassembly detection using Stash: A data structure based on stochastic tile hashing”. This file contains Supplementary Tables 1–19, Supplementary Figures 1–4, Supplementary Methods 1–3, Supplementary Examples 1–2, and Supplementary Results 1–6.
(DOCX)

## References

[1] Brown D, Morgenstern B. Algorithms in bioinformatics. Berlin: Springer; 2014. doi: 10.1007/978-3-662-44753-6

[2] Bloom BH. Space/time trade-offs in hash coding with allowable errors. Commun ACM. 1970;13(7):422–6. doi: 10.1145/362686.362692

[3] Chu J, Mohamadi H, Erhan E, Tse J, Chiu R, Yeo S, et al. Mismatch-tolerant, alignment-free sequence classification using multiple spaced seeds and multiindex Bloom filters. Proc Natl Acad Sci U S A. 2020;117(29):16961–8. doi: 10.1073/pnas.1903436117 32641514 PMC7382288

[4] Bender MA, Farach-Colton M, Johnson R, Kuszmaul BC, Medjedovic D, Montes P, et al. Don’t thrash: how to cache your hash on flash. Proc VLDB Endow. 2012;5(11):1627–37. doi: 10.14778/2350229.2350275

[5] Pandey P, Bender MA, Johnson R, Patro R. A general-purpose counting filter: making every bit count. Proceedings of the 2017 ACM International Conference on Management of Data (SIGMOD); 2017. p. 775–87. 10.1145/3035918.3035963

[6] Fan B, Andersen DG, Kaminsky M, Mitzenmacher MD. Cuckoo filter: practically better than Bloom. Proceedings of the 10th ACM International Conference on Emerging Networking Experiments and Technologies (CoNEXT); 2014. p. 75–88. 10.1145/2674005.2674994

[7] Marchet C, Lecompte L, Limasset A, Bittner L, Peterlongo P. A resource-frugal probabilistic dictionary and applications in bioinformatics. Discrete Appl Math. 2020;274:92–102. doi: 10.1016/j.dam.2018.03.035

[8] Abo RP, Ducar M, Garcia EP, Thorner AR, Rojas-Rudilla V, Lin L, et al. BreaKmer: detection of structural variation in targeted massively parallel sequencing data using kmers. Nucleic Acids Res. 2015;43(3):e19. doi: 10.1093/nar/gku1211 25428359 PMC4330340

[9] Wang Y, Xue H, Pourcel C, Du Y, Gautheret D. 2-kupl: mapping-free variant detection from DNA-seq data of matched samples. BMC Bioinform. 2021;22(1):304. doi: 10.1186/s12859-021-04185-6 34090332 PMC8180056

[10] Hippe K, Gbenro S, Cao R. ProLanGO2: Protein function prediction with ensemble of encoder-decoder networks. Proceedings of the 11th ACM International Conference on Bioinformatics, Computational Biology and Health Informatics (BCB); 2020. p. 1–6. 10.1145/3388440.3414701

[11] Qin Y-F, Wang C-H, Yu X-Q, Zhu J, Liu T-G, Zheng X-Q. Predicting protein structural class by incorporating patterns of over-represented k-mers into the general form of Chou’s PseAAC. Protein Pept Lett. 2012;19(4):388–97. doi: 10.2174/092986612799789350 22316305

[12] Marchet C, Boucher C, Puglisi SJ, Medvedev P, Salson M, Chikhi R. Data structures based on k-mers for querying large collections of sequencing data sets. Genome Res. 2021;31(1):1–12. doi: 10.1101/gr.260604.119 33328168 PMC7849385

[13] Mian E, Petrucci E, Pizzi C, Comin M. Efficient hashing of multiple spaced seeds with application. BIOINFORMATICS; 2023. p. 155–62. 10.5220/0011632900003414 39320990

[14] Kazemi P, Wong J, Nikolić V, Mohamadi H, Warren RL, Birol I. ntHash2: recursive spaced seed hashing for nucleotide sequences. Bioinformatics. 2022;38(20):4812–3. doi: 10.1093/bioinformatics/btac564 36000872 PMC9563681

[15] Mohamadi H, Chu J, Vandervalk BP, Birol I. ntHash: recursive nucleotide hashing. Bioinformatics. 2016;32(22):3492–4. doi: 10.1093/bioinformatics/btw397 27423894 PMC5181554

[16] Dida F, Yi G. Empirical evaluation of methods for de novo genome assembly. PeerJ Comput Sci. 2021;7:e636. doi: 10.7717/peerj-cs.636 34307867 PMC8279138

[17] Fitz-Gibbon S, Hipp AL, Pham KK, Manos PS, Sork VL. Phylogenomic inferences from reference-mapped and de novo assembled short-read sequence data using RADseq sequencing of California white oaks (*Quercus* section *Quercus*). Genome. 2017;60(9):743–55. doi: 10.1139/gen-2016-0202 28355490

[18] Warren RL, Keeling CI, Yuen MMS, Raymond A, Taylor GA, Vandervalk BP, et al. Improved white spruce (*Picea glauca*) genome assemblies and annotation of large gene families of conifer terpenoid and phenolic defense metabolism. Plant J. 2015;83(2):189–212. doi: 10.1111/tpj.12886 26017574

[19] Park ST, Kim J. Trends in next-generation sequencing and a new era for whole genome sequencing. Int Neurourol J. 2016;20(Suppl 2):S76-83. doi: 10.5213/inj.1632742.371 27915479 PMC5169091

[20] Hunt M, Kikuchi T, Sanders M, Newbold C, Berriman M, Otto TD. REAPR: a universal tool for genome assembly evaluation. Genome Biol. 2013;14(5):R47. doi: 10.1186/gb-2013-14-5-r47 23710727 PMC3798757

[21] Walker BJ, Abeel T, Shea T, Priest M, Abouelliel A, Sakthikumar S, et al. Pilon: an integrated tool for comprehensive microbial variant detection and genome assembly improvement. PLoS One. 2014;9(11):e112963. doi: 10.1371/journal.pone.0112963 25409509 PMC4237348

[22] Jackman SD, Coombe L, Chu J, Warren RL, Vandervalk BP, Yeo S, et al. Tigmint: correcting assembly errors using linked reads from large molecules. BMC Bioinform. 2018;19(1):393. doi: 10.1186/s12859-018-2425-6 30367597 PMC6204047

[23] Peona V, Blom MPK, Xu L, Burri R, Sullivan S, Bunikis I, et al. Identifying the causes and consequences of assembly gaps using a multiplatform genome assembly of a bird-of-paradise. Mol Ecol Resour. 2021;21(1):263–86. doi: 10.1111/1755-0998.13252 32937018 PMC7757076

[24] Coombe L, Li JX, Lo T, Wong J, Nikolic V, Warren RL, et al. LongStitch: high-quality genome assembly correction and scaffolding using long reads. BMC Bioinform. 2021;22(1):534. doi: 10.1186/s12859-021-04451-7 34717540 PMC8557608

[25] Bao E, Song C, Lan L. ReMILO: reference assisted misassembly detection algorithm using short and long reads. Bioinformatics. 2018;34(1):24–32. doi: 10.1093/bioinformatics/btx524 28961789

[26] Chen Y, Zhang Y, Wang AY, Gao M, Chong Z. Accurate long-read de novo assembly evaluation with Inspector. Genome Biol. 2021;22(1):312. doi: 10.1186/s13059-021-02527-4 34775997 PMC8590762

[27] Zhang Y, Lu H-W, Ruan J. GAEP: a comprehensive genome assembly evaluating pipeline. J Genet Genomics. 2023;50(10):747–54. doi: 10.1016/j.jgg.2023.05.009 37245652

[28] Wenger AM, Peluso P, Rowell WJ, Chang P-C, Hall RJ, Concepcion GT, et al. Accurate circular consensus long-read sequencing improves variant detection and assembly of a human genome. Nat Biotechnol. 2019;37(10):1155–62. doi: 10.1038/s41587-019-0217-9 31406327 PMC6776680

[29] Kolmogorov M, Yuan J, Lin Y, Pevzner PA. Assembly of long, error-prone reads using repeat graphs. Nat Biotechnol. 2019;37(5):540–6. doi: 10.1038/s41587-019-0072-8 30936562

[30] Shafin K, Pesout T, Lorig-Roach R, Haukness M, Olsen HE, Bosworth C, et al. Nanopore sequencing and the Shasta toolkit enable efficient de novo assembly of eleven human genomes. Nat Biotechnol. 2020;38(9):1044–53. doi: 10.1038/s41587-020-0503-6 32686750 PMC7483855

[31] Coombe L, Warren RL, Wong J, Nikolic V, Birol I. ntLink: a toolkit for de novo genome assembly scaffolding and mapping using long reads. Curr Protoc. 2023;3(4):e733. doi: 10.1002/cpz1.733 37039735 PMC10091225

[32] Gurevich A, Saveliev V, Vyahhi N, Tesler G. QUAST: quality assessment tool for genome assemblies. Bioinformatics. 2013;29(8):1072–5. doi: 10.1093/bioinformatics/btt086 23422339 PMC3624806

[33] Ewing B, Green P. Base-calling of automated sequencer traces using phred. II. Error probabilities. Genome Res. 1998;8(3):186–94. doi: 10.1101/gr.8.3.186 9521922

[34] Ewing B, Hillier L, Wendl MC, Green P. Base-calling of automated sequencer traces using phred. I. Accuracy assessment. Genome Res. 1998;8(3):175–85. doi: 10.1101/gr.8.3.175 9521921

