# Supplementary material for: Genome misassembly detection using Stash: A data structure based on stochastic tile hashing
Source: PLoS One. 2026 Jul 13;21(7):e0333729. doi: 10.1371/journal.pone.0333729 (PMC13362140; doi:10.1371/journal.pone.0333729)
Supplement: S1 File — This file contains Supplementary Tables 1–19, Supplementary Figures 1–4, Supplementary Methods 1–3, Supplementary Examples 1–2, and Supplementary Results 1–6. (DOCX) [file pone.0333729.s001.docx]

Supplementary Information for:
Genome misassembly detection using Stash: A data structure based on stochastic tile hashing

Armaghan Sarvar^1,2^, Lauren Coombe^2^, and Inanc Birol^1,2^

^1^University of British Columbia, Vancouver, Canada and

^2^BC Cancer, Vancouver, Canada.

Table of Contents

[Supplementary Table 1. Long read sequencing read set used for genome assembly benchmarks and Stash algorithm visualization 3](#_Toc160990650)

[Supplementary Table 2. Symmetrical spaced seed patterns used during the experiments. 3](#_Toc160990652)

[Supplementary Table 3. The assemblies used for the misassembly detection experiment. 3](#_Toc160990651)

[Supplementary Table 4. Percentage of samples that fall above the 90% prediction interval of the unrelated distribution. 4](#_Toc160990653)

[Supplementary Table 5. Percentage of samples that fall above the 99% prediction interval of the unrelated distribution. 4](#_Toc160990654)

[Supplementary Table 6. Comparison of multiple misassembly correction methods on the Flye assembly. 5](#_Toc160990655)

[Supplementary Table 7. Comparison of multiple misassembly correction methods on the Flye assembly followed by scaffolding. 5](#_Toc160990656)

[Supplementary Table 8. Comparison of multiple misassembly correction methods on the Shasta assembly. 6](#_Toc160990661)

[Supplementary Table 9. Comparison of multiple misassembly correction methods on the Shasta assembly followed by scaffolding. 6](#_Toc160990662)

Supplementary Table 10. Comparison of multiple misassembly correction methods on the ONT Flye assembly…………………………...……..17
Supplementary Table 11. Comparison of multiple misassembly correction methods on the ONT Flye assembly followed by scaffolding…….....18
Supplementary Table 12. Comparison of multiple misassembly correction methods on the C. elegans ASM1813679v1 assembly……………….18
Supplementary Table 13. Comparison of multiple misassembly correction methods on the C. elegans ASM1813679v1 assembly followed by scaffolding……………………………………………………………………………………………………………………………………………19

Supplementary Table 14. Sensitivity of StashCut to reduced read depth via read downsampling……………………………………….………….21
Supplementary Table 15. Spatial overlap of misassembly breakpoints on the Flye assembly………………………………………………….…...23
Supplementary Table 16. Spatial overlap of misassembly breakpoints on the Shasta assembly……………………………………………………23
Supplementary Table 17. Extensive misassembly event rates with confidence intervals and rate ratios (pre/post scaffolding)… ………………...24
Supplementary Table 18. Misassembly correction on human mtDNA……………………………………………………………………………...25
Supplementary Table 19. Extensive misassembly breakpoints by genomic context………………………………………………………….….…26
[Supplementary Fig. 1. Effectiveness of the number of matches metric defined for a pair of Stash windows. 7](file:////Users/armaghansarvar/Downloads/Stash_Supplementary_Modified%20(1).docx#_Toc160990667)

[Supplementary Fig. 2. Parameter sweep of *StashCut* performed on the Shasta assembly. 7](file:////Users/armaghansarvar/Downloads/Stash_Supplementary_Modified%20(1).docx#_Toc160990668)

[Supplementary Fig. 3. Precision-recall curve from running *StashCut* on the Flye assembly. 8](file:////Users/armaghansarvar/Downloads/Stash_Supplementary_Modified%20(1).docx#_Toc160990669)

[Supplementary Fig. 4. Precision-recall curve from running *StashCut* on the Shasta assembly. 8](file:////Users/armaghansarvar/Downloads/Stash_Supplementary_Modified%20(1).docx#_Toc160990670)

[Supplementary Method 1. Time complexities 9](#_Toc160990671)

[Supplementary Method 2. Computing the *Stash-cut* threshold 10](#_Toc160990680)

[Supplementary Method 3. Custom *Stash-cut* metrics 11](#_Toc160990683)

[Supplementary Example 1. Insertion into Stash 13](#_Toc160990684)

[Supplementary Example 2. Querying Stash 15](#_Toc160990685)

[Supplementary Results 1. Evaluation of StashCut on ONT Q20+ human genome assemblies at the contig and scaffold levels. 18](#_Toc160990686)

Supplementary Results 2. Evaluating *StashCut* on PacBio RS II C. elegans reads and assembly. 19
Supplementary Results 3. Sensitivity to reduced read depth via read downsampling (fixed assembly)………………………………………….....20
Supplementary Results 4. Spatial overlap analysis of misassembly breakpoints…………………..……………………. ……………..…….….....22
Supplementary Results 5 Evaluation on human mitochondrial genome assembly………………………………………………………………….25
Supplementary Results 6. Distribution of misassembly corrections across genomic contexts………………………………………………...……26

Supplementary Table 1. Long read sequencing read set used for genome assembly benchmarks and Stash algorithm visualization

Supplementary Table 3. The assemblies used for the misassembly detection experiment. Note that this information was generated by QUAST, and only represents contigs longer than 3,000 bp. The software versions and input parameters are as follows:  ntLink: 1.3.5, k=80 w=250; Shasta: 0.10.0, conf=HiFi-Oct2021.conf; Flye: 2.9-b1768, conf=pacbio-hifi, G=3G.

Supplementary Table 2. Symmetrical spaced seed patterns used during the experiments.

| Assembly | Total length (bp) | Largest Contig (bp) | NG50 (bp) | NGA50 (bp) | Extensive Misassemblies | Local  Misassemblies | Relocations | Translocations | Inversions |
| --- | --- | --- | --- | --- | --- | --- | --- | --- | --- |
| Flye | 3,213,468,360 | 36,496,138 | 5,181,338 | 4,840,877 | 9,544 | 11,686 | 6,726 | 2,769 | 49 |
| Scaffolded Flye | 3,237,006,440 | 74,691,217 | 17,474,991 | 13,943,923 | 9,745 | 11,660 | 6,861 | 2,833 | 51 |
| Shasta | 3,054,820,697 | 138,091,504 | 31,604,298 | 25,477,820 | 7,071 | 11,003 | 5,711 | 1,312 | 48 |
| Scaffolded Shasta | 3,053,620,707 | 138,119,290 | 37,344,674 | 27,076,873 | 7,683 | 10,322 | 6,038 | 1,588 | 57 |

| 1 | 1 | 0 | 1 | 1 | 0 | 1 | 1 | 1 | 0 | 1 | 1 | 1 | 1 | 1 | 1 | 0 | 1 | 1 | 1 | 0 | 1 | 1 | 0 | 1 | 1 |
| --- | --- | --- | --- | --- | --- | --- | --- | --- | --- | --- | --- | --- | --- | --- | --- | --- | --- | --- | --- | --- | --- | --- | --- | --- | --- |
| 1 | 0 | 1 | 1 | 1 | 1 | 0 | 0 | 1 | 1 | 1 | 1 | 1 | 1 | 1 | 1 | 1 | 1 | 0 | 0 | 1 | 1 | 1 | 1 | 0 | 1 |
| 1 | 1 | 1 | 0 | 1 | 1 | 1 | 1 | 0 | 1 | 1 | 0 | 1 | 1 | 0 | 1 | 1 | 0 | 1 | 1 | 1 | 1 | 0 | 1 | 1 | 1 |
| 1 | 0 | 1 | 1 | 0 | 1 | 1 | 1 | 1 | 1 | 1 | 1 | 0 | 0 | 1 | 1 | 1 | 1 | 1 | 1 | 1 | 0 | 1 | 1 | 0 | 1 |

| Species | Cell line/Strain | Fold  Coverage | N50 Length (bp) | Accession/Source |
| --- | --- | --- | --- | --- |
| *H. sapiens* | NA24385 | 28 | 13,480 | https://www.ncbi.nlm.nih.gov/sra/SRX5327410 |

Supplementary Table 4. Percentage of samples that fall above the 90% prediction interval of the unrelated distribution. As Delta is increased, the match distribution of a pair of Stash windows will become gradually less distinguishable from the matches distribution of two random windows. The values in this table are derived from Figure 3 of the manuscript.

| *Delta* (bp) | Above 90%  Prediction Interval (%) | *Delta* (bp) | Above 90%  Prediction Interval (%) |
| --- | --- | --- | --- |
| 1 | 91.8 | 256 | 87.8 |
| 2 | 90.9 | 512 | 86.6 |
| 4 | 89.9 | 1,024 | 83.9 |
| 8 | 90.9 | 2,048 | 81.7 |
| 16 | 91.8 | 4,096 | 70.4 |
| 32 | 90.2 | 8,192 | 38.1 |
| 64 | 88.4 | 16,384 | 17.8 |
| 128 | 89.8 |  |  |

Supplementary Table 5. Percentage of samples that fall above the 99% prediction interval of the unrelated distribution. As Delta is increased, the match distribution of a pair of Stash windows will become gradually less distinguishable from the matches distribution of two random windows. The values in this table are derived from Figure 3 of the manuscript.

| *Delta* (bp) | Above 99%  Prediction Interval (%) | *Delta* (bp) | Above 99%  Prediction Interval (%) |
| --- | --- | --- | --- |
| 1 | 78.4 | 256 | 74.0 |
| 2 | 79.7 | 512 | 70.5 |
| 4 | 76.5 | 1,024 | 66.5 |
| 8 | 76.4 | 2,048 | 57.3 |
| 16 | 78.6 | 4,096 | 38.8 |
| 32 | 77.3 | 8,192 | 10.7 |
| 64 | 77.0 | 16,384 | 6.6 |
| 128 | 76.0 |  |  |

| Assembly | Total length (bp) | Largest Contig (bp) | NG50 (bp) | NGA50 (bp) | Extensive Misassemblies | Local  Misassemblies | Relocations | Translocations | Inversions |
| --- | --- | --- | --- | --- | --- | --- | --- | --- | --- |
| Flye | 3,213,468,360 | 36,496,138 | 5,181,338 | 4,840,877 | 9,544 | 11,686 | 6,726 | 2,769 | 49 |
| Flye + *StashCut* (v1.2.0) | 3,213,336,421 | 36,496,138 | 5,133,808 | 4,840,877 | 8,326 | 12,140 | 6,183 | 2,096 | 47 |
| Flye + Inspector (v1.0.1) | 3,212,309,565 | 36,496,138 | 5,181,363 | 4,840,933 | 9,569 | 11,651 | 6,758 | 2,763 | 48 |
| Flye + Tigmint-long (v1.2.2) | 3,211,270,058 | 28,387,839 | 3,813,289 | 3,768,849 | 9,339 | 11,651 | 6,656 | 2,629 | 54 |

Supplementary Table 7. **Comparison of multiple misassembly correction methods on the Flye assembly followed by scaffolding.** StashCut reduces the number of extensive misassemblies relative to the baseline scaffolded assembly, with a trade-off in contiguity relative to Tigmint-long. The first row of the table is the QUAST evaluation of the Flye scaffold (Flye + ntLink) while other rows represent the evaluation on the output of StashCut, Inspector, and Tigmint-long misassembly correction methods applied to the Flye assembly followed by the same ntLink scaffolding step.

Supplementary Table 6. Comparison of multiple misassembly correction methods on the Flye assembly. StashCut reduces the number of extensive misassemblies compared to the other tools, without sacrificing contiguity. The first row of the table is the QUAST evaluation of the Flye assembly while other rows represent the evaluation on the output of StashCut, Inspector, and -t-long misassembly correction methods performed on the Flye assembly.

| Assembly | Total length (bp) | Largest Contig (bp) | NG50 (bp) | NGA50 (bp) | Extensive Misassemblies | Local  Misassemblies | Relocations | Translocations | Inversions |
| --- | --- | --- | --- | --- | --- | --- | --- | --- | --- |
| Flye + ntLink | 3,237,006,440 | 74,691,217 | 17,474,991 | 13,943,923 | 9,745 | 11,660 | 6,861 | 2,833 | 51 |
| Flye + *StashCut* + ntLink | 3,236,252,577 | 74,691,217 | 17,966,166 | 14,181,592 | 9,003 | 11,510 | 6,441 | 2,508 | 54 |
| Flye + Inspector + ntLink | 3,236,539,115 | 74,691,388 | 18,214,108 | 14,624,442 | 9,756 | 11,641 | 6,908 | 2,798 | 50 |
| Flye + Tigmint-long + ntLink | 3,233,215,096 | 111,634,790 | 19,420,865 | 16,500,644 | 9,489 | 11,550 | 6,752 | 2,678 | 59 |

Supplementary Table 8. Comparison of multiple misassembly correction methods on the Shasta assembly. The first row of the table is the QUAST evaluation of the Shasta assembly while other rows represent the evaluation on the output of StashCut, Inspector, and Tigmint-long misassembly correction methods performed on the Shasta assembly.

| Assembly | Total length (bp) | Largest Contig (bp) | NG50 (bp) | NGA50 (bp) | Extensive Misassemblies | Local  Misassemblies | Relocations | Translocations | Inversions |
| --- | --- | --- | --- | --- | --- | --- | --- | --- | --- |
| Shasta | 3,054,820,697 | 138,091,504 | 31,604,298 | 25,477,820 | 7,071 | 11,003 | 5,711 | 1,312 | 48 |
| Shasta + *StashCut* | 3,054,406,420 | 138,091,504 | 31,418,895 | 24,033,390 | 6,683 | 11,058 | 5,425 | 1,215 | 43 |
| Shasta + Inspector | 3,041,329,116 | 138,091,305 | 31,604,248 | 25,477,833 | 6,922 | 10,695 | 5,594 | 1,284 | 44 |
| Shasta + Tigmint-long | 3,053,408,806 | 121,366,450 | 19,079,701 | 16,745,679 | 7,020 | 10,965 | 5,626 | 1,346 | 48 |

Supplementary Table 9. Comparison of multiple misassembly correction methods on the Shasta assembly followed by scaffolding. The first row of the table is the QUAST evaluation of the Shasta scaffold while other rows represent the evaluation on the output of StashCut, Inspector, and Tigmint-long misassembly correction methods performed on the Shasta scaffold.

| Assembly | Total length (bp) | Largest Contig (bp) | NG50 (bp) | NGA50 (bp) | Extensive Misassemblies | Local  Misassemblies | Relocations | Translocations | Inversions |
| --- | --- | --- | --- | --- | --- | --- | --- | --- | --- |
| Shasta + ntLink | 3,053,620,707 | 138,119,290 | 37,344,674 | 27,076,873 | 7,683 | 10,322 | 6,038 | 1,588 | 57 |
| Shasta + *StashCut* + ntLink | 3,053,102,277 | 138,119,290 | 37,344,674 | 27,076,873 | 7,420 | 10,348 | 5,812 | 1,553 | 55 |
| Shasta +  Inspector + ntLink | 3,038,565,682 | 138,154,468 | 34,476,778 | 26,805,010 | 7,428 | 10,078 | 5,899 | 1,471 | 58 |
| Shasta + Tigmint-long + ntLink | 3,052,792,046 | 138,119,529 | 37,344,834 | 26,804,070 | 7,681 | 10,318 | 6,032 | 1,594 | 55 |


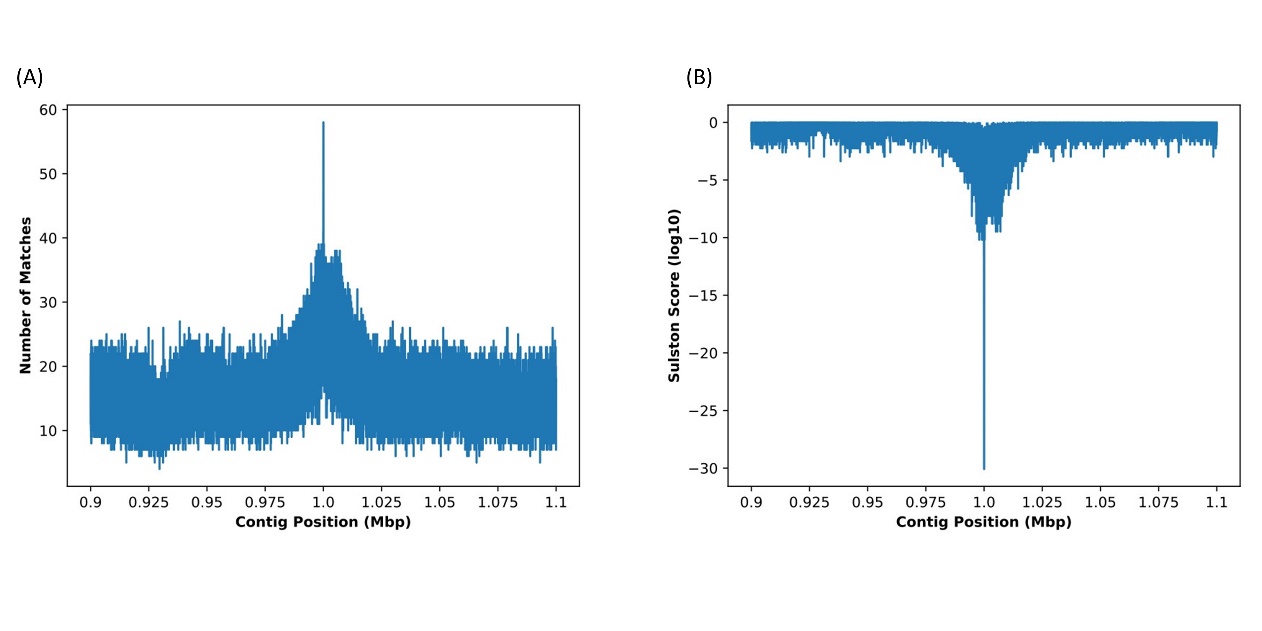


Supplementary Fig. 1. Effectiveness of the number of matches metric defined for a pair of Stash windows. The matches signal when Stash is queried on one window fixed at position 1,000,000 and another window sliding from position 900,000 to 1,100,000 of the reference human genome GRCh38 chromosome 21. The noticeable peak in the number of matches signal represents the query when the two windows have overlapping frames. In this experiment, Stash was filled with HiFi sequencing reads from chromosome 21 of the human individual NA24385.


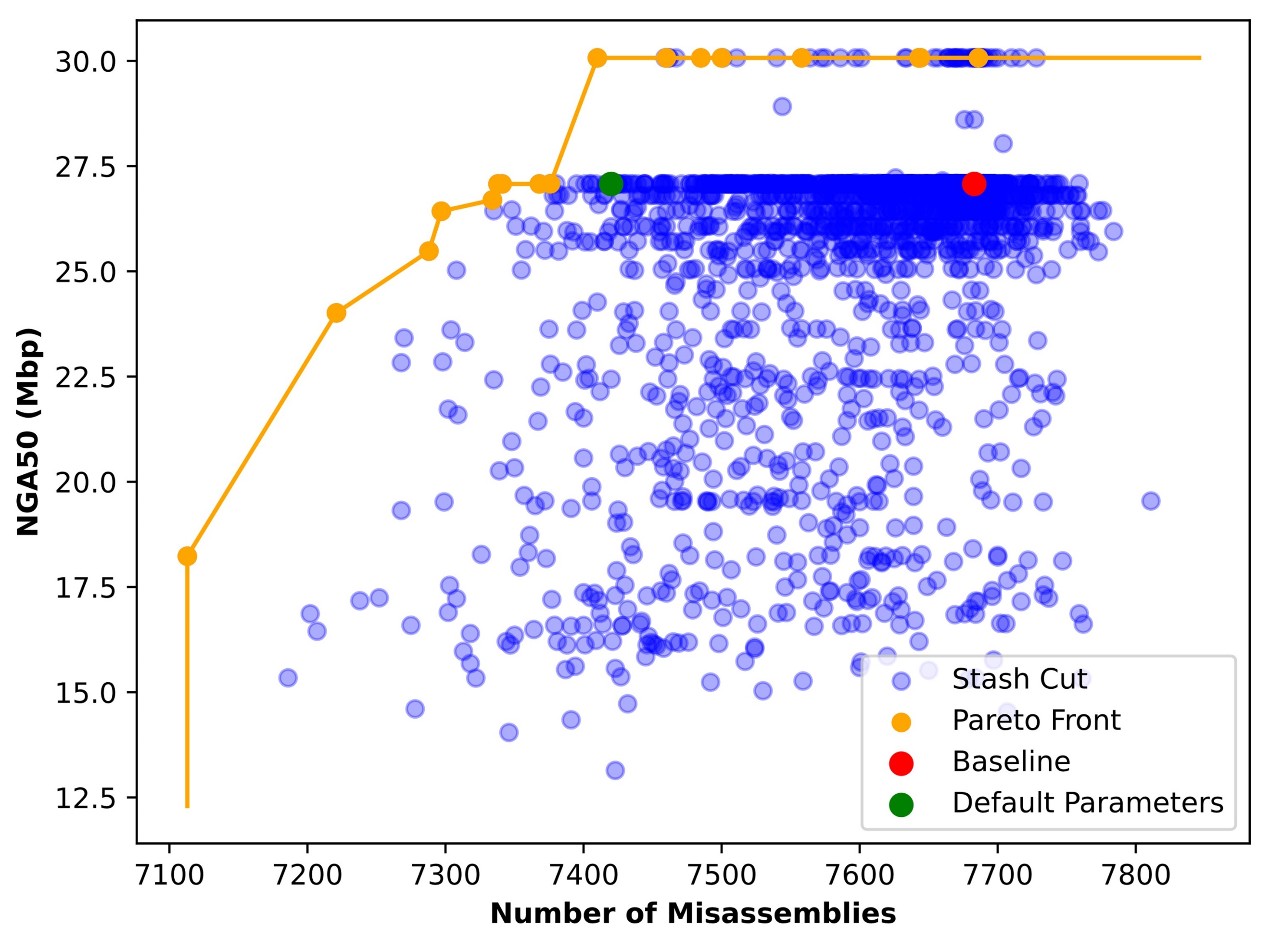


Supplementary Fig. 2. Parameter sweep of StashCut performed on the Shasta assembly. Results were obtained using QUAST on the ntLink scaffold of the assembly. As displayed by the pareto frontier, higher quality cuts should result in lower number of misassemblies and higher NGA50. The figure displays the evaluation of 3,125 different configurations of StashCut.


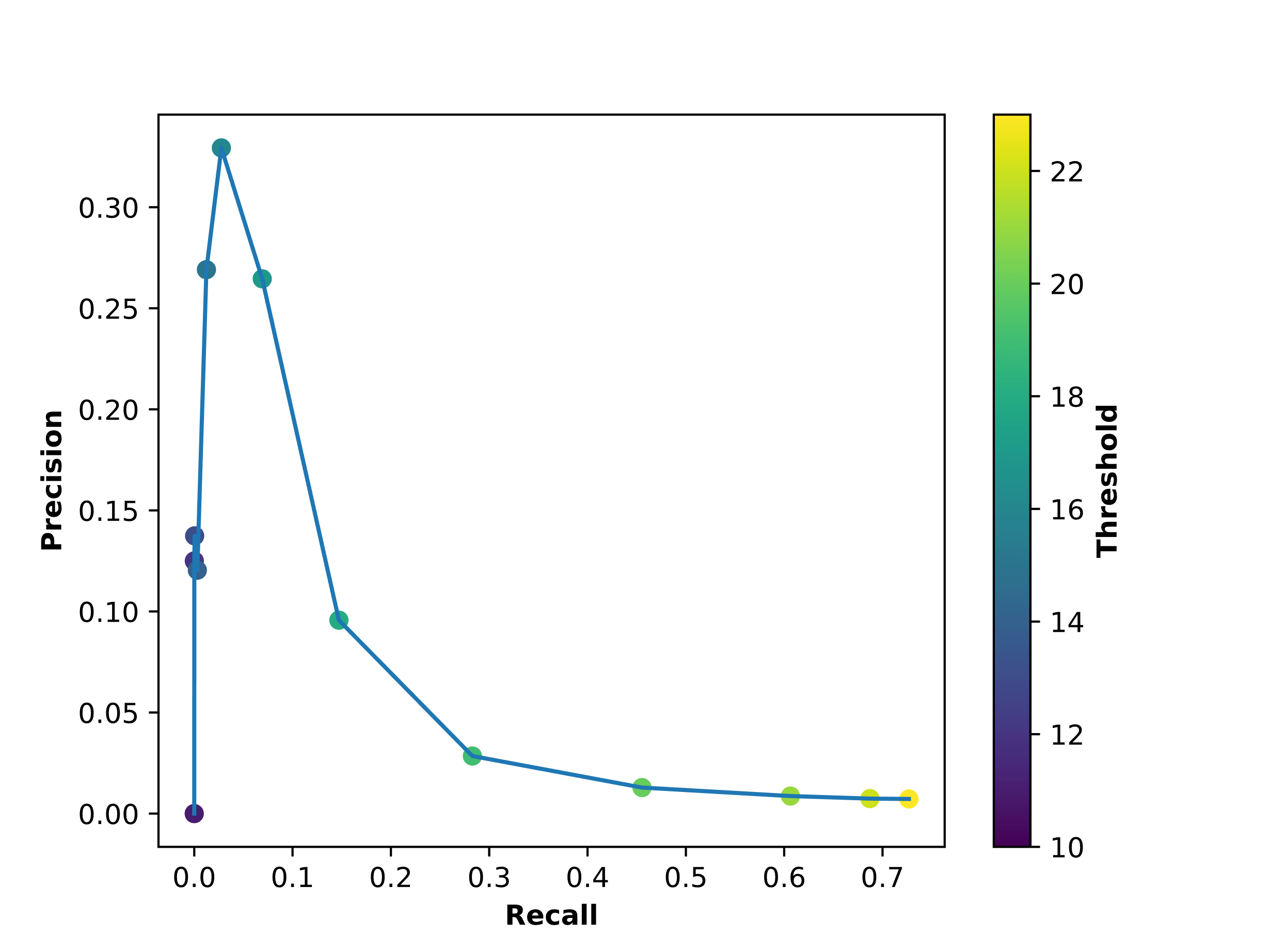


Supplementary Fig. 3. Precision-recall curve from running StashCut on the Flye assembly. Data points represent our custom precision and recall metrics of StashCut configurations that only differ in the threshold parameter. Other parameters are equal to the default parameter set of StashCut. Note that this is not a standard PR curve due to the custom StashCut definitions of precision and recall.


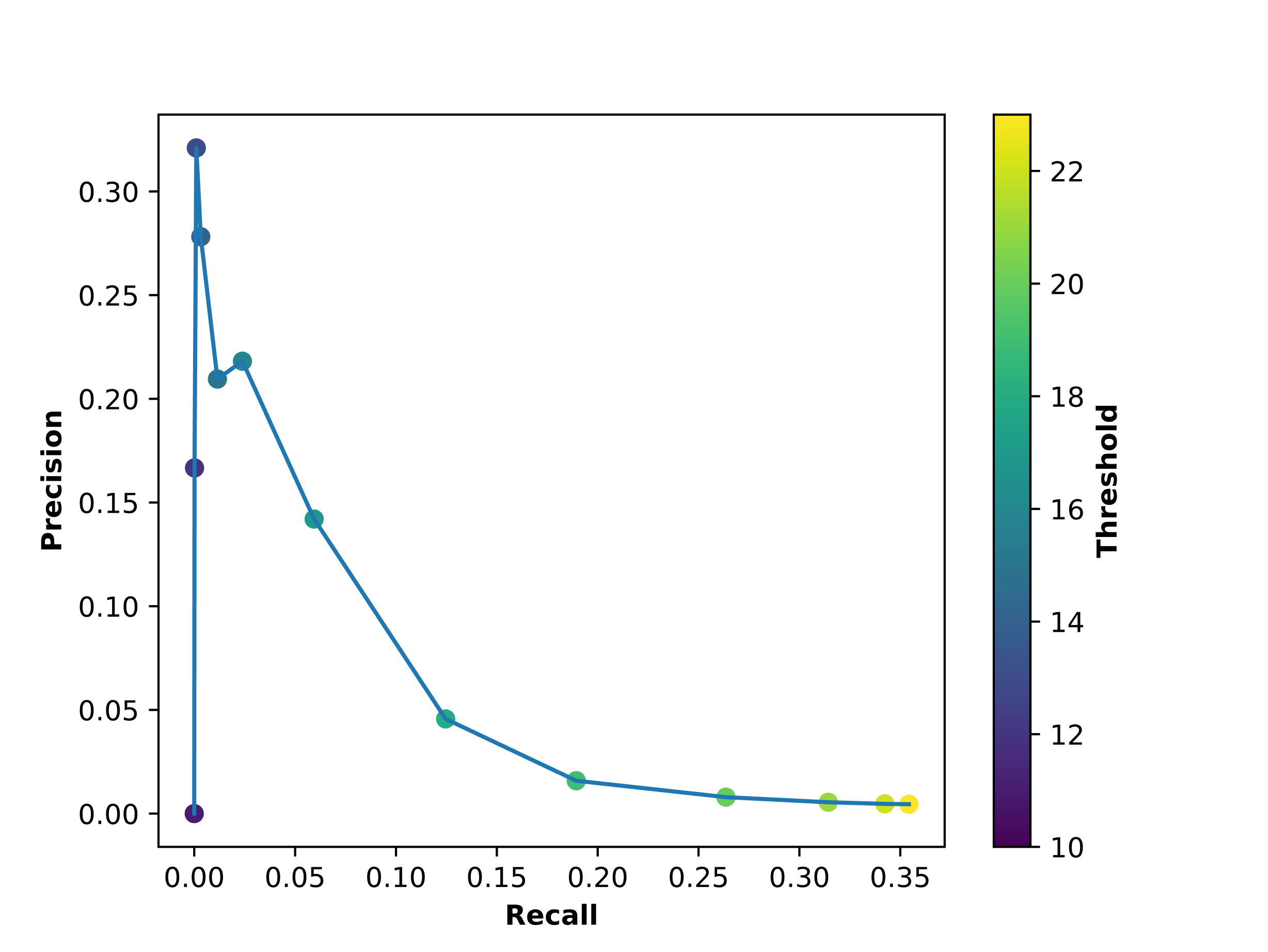


Supplementary Fig. 4. Precision-recall curve from running StashCut on the Shasta assembly. Data points represent our custom precision and recall metrics of StashCut configurations that only differ in the threshold parameter. Other parameters are equal to the default parameter set of StashCut. Note that this is not a standard PR curve due to the custom StashCut definitions of precision and recall.

Supplementary Method 1

In this section we explain the time complexities provided in the manuscript.

The insertion of a single sequence in Stash is actually *h* different insertions, where *h* is the number of spaced seed patterns or hash values. We require the sequence to be hashed *h* times, which in isolation requires a time complexity of $O\left( h\times length \right)$, assuming the hash function has linear complexity. Next, we need to combine the hash functions to create tile indices. Each index takes $O(h)$ time, and we have *h* indices, resulting in $O\left( h^{2} \right)$. The last step is the insertion itself, which simply takes $O(h)$ as we perform *h* insertions. Overall, the insertion complexity of an isolated sequence would be as follows.

$$O(h\times length+h^{2})$$

If the user is interested in inserting a long sequence in Stash, they can slide over the sequence and extract a set of subsequences with fixed lengths. In this case, rolling hashes could be utilized to reduce the computation. Given *n* long sequences with average length of *l*, the sequences can be inserted into the Stash with the following time complexity. Note that a sequence of length *l* has $\left( l-k+1 \right)$ *k*-mers, but we exclude *k* from the time complexity as Stash is primarily proposed for long reads which are significantly longer than *k* base-pairs.

$$O\left( h^{2}ln \right)$$

At query time, two sequences of length *k* can be given to Stash to determine whether they originated from the same sequence IDs. We first hash both in $O(hk)$ time, then access the corresponding Stash frames and count the number of matches in $O(h^{2}C)$, where *C* is the number of tiles in a Stash frame row and *h* is the number of rows in a Stash frame. Specifically, every tile in a Stash frame $\left( C\times h \right)$, needs to be compared to all tiles in the same column of the other Stash frame $(h)$. Therefore, the following is the time complexity of computing the number of matches for Stash frames of the two input sequences.

$$O\left( h^{2}C+hk \right)$$

When comparing two Stash windows rather than Stash frames, we would require $f^{2}$ number of matches extracted, where *f* is the number of Stash frames in each Stash window.

$$O(f^{2}h^{2}C+fhk)$$

Supplementary Method 2

The expected number of matches between two Stash windows can be computed as follows.

Probability of a Stash tile matching another:

For tiles storing t₂ bits, there are 2^(t₂) possible values. Assuming uniform distribution, the probability that a specific tile from frame A matches a specific tile in the same column of frame B is:

$$\pi= 2^{-t_{2}}$$

Probability that a tile from frame A matches at least one of h tiles in the same column of frame B:

$$q = 1 - \left( 1 - \pi\right)^{h}$$

Probability of having exactly m matches between two Stash frames of T = hC total tile positions:

$$P\left( M=m \right)=\binom{T}{m}\left( q \right)^{m}\left( 1-q \right)^{T-m}$$

Probability of having at least m matches between two Stash frames:

$$p(M\geq m)=\sum_{i=m}^{T} \binom{T}{i}\left( q \right)^{i}\left( 1-q \right)^{T-i}$$

Expected match count for unrelated windows with $f$ frames:

When comparing two windows each containing $f$frames, we compute $f^{2}$pairwise match counts and take the maximum. Under the null hypothesis, each pairwise match count follows M ~ Binomial(T, q). The distribution of the maximum across $f^{2}$comparisons is difficult to characterize analytically due to dependencies between frame pairs (frames within a window share rows and are affected by saturation). Therefore, we use empirical calibration: we estimate the null distribution by comparing unrelated windows (from different chromosomes or distant genomic positions) and derive thresholds from quantiles of this empirical distribution.

Supplementary Method 3

For the task of misassembly detection, we have defined custom evaluation metrics to provide an immediate evaluation of *StashCut* without the need to run an assembly evaluation tool on the corrected assembly. Our metrics require the true misassembly locations of the input assembly, which we obtain using QUAST. Each extensive misassembly of QUAST is reported using the extents of the two flanking sequences that are incorrectly connected in the input assembly. We define the true position of the misassembly to be the location in the middle of the left flanking sequence’s right-end and the right flanking sequence’s left-end, and the length of the misassembly to be equal to the gap between them. Using the length, we can expect drops in the matches signal centered around the misassembly position for a specific Stash window configuration as follows.

$$Drop Region=2l_{ss}+\Delta+l_{m}-1$$

where $l_{ss}$ is the spaced seed length, $\Delta$ is the distance between the two sliding windows, and $l_{m}$ is the length of the misassembly. True positive (TP) is then defined as a cut that lies in the expected drop region around the misassembly position. We only consider one TP per QUAST misassembly which contributes to well-defined false negatives (FN). If we consider all QUAST misassemblies as positive data points, TP corresponds to misassmblies that *StashCut* is able to detect, while FN corresponds to drop regions that elude detection. As negative data points are not clearly defined in the misassembly detection problem, the true negatives (TN) measure is undefined. We defined FP as the cuts that are not TPs.

With TP, FP, and FN defined, we are able to calculate precision and recall for a *StashCut* corrected assembly, where the former describes how precisely we are choosing our cut locations (TP over TP + FP) and the latter measures what fraction of misassemblies we are able to detect (TP over TP + FN).

To quantify uncertainty for the tool-to-tool comparisons reported using QUAST, we model extensive misassemblies as event counts under a Poisson process with exposure defined by the assembled length (in gigabases) and report rates per assembled gigabase (events/Gbp) with 95% confidence intervals under a Poisson model (Garwood interval). To compare methods, we report Poisson rate ratios (RR) relative to the baseline assembly for each dataset (i.e., comparing misassembly rates rather than raw counts, using assembled length as the offset), with 95% confidence intervals computed on the log scale using variance $\frac{1}{k_{1}}+\frac{1}{k_{0}}$​, where *k* denotes the extensive misassembly count for the method and baseline, respectively. Two-sided p-values are computed using a Wald test on $\log\left( RR \right)$ under the null hypothesis log(RR)=0 (equivalently, RR=1). These statistics are summarized in Supplementary Table 17.

Supplementary Example 1

This section illustrates how a sequence can be inserted into the Stash data structure.

Given Sequence:

AACGTTGATTCCGA Sequence ID = “My Test Sequence”

Set of Spaced Seeds:

10101100110101

11010011001011

10101011010101

11010100101011

First, the spaced seed patterns are applied over the sequence, resulting in the following *k*-mers, which are then hashed to produce integer values.

ACTTTTCA => Hash: 388

AAGGACGA => Hash: 5

ACTGATCA => Hash: 1009

AAGTTCGA => Hash: 217

Each *k*-mer corresponds to an insertion in the Stash data structure, where the row of the insertion is determined as the remainder of the *k*-mer hash value by the number of Stash rows. For example, given a Stash with 256 rows, the following Stash rows will be modified.

388 mod 256 = 132

5 mod 256 = 5

1009 mod 256 = 241

217 mod 256 = 217

Determining the column or the value of the insertion is a two-step process. We first need to transform the above hash values into a tile index, then use that index to retrieve the column or the value of the insertion.

The transformation applied on one hash value will generate a tile index by combining all other hash values. Asuming we have 8 tiles to address, below is the transformation.

132 In Binary => 1000 0100 => Combine the first left-most bits of other hashes => 011

5 In Binary => 0000 0101 => Combine the second left-most bits of other hashes => 011

241 In Binary => 1111 0001 => Combine the third left-most bits of other hashes => 000

217 In Binary => 1101 1001 => Combine the fourth left-most bits of other hashes => 001

The tile indices can now be used to obtain the column and the value of the insertion. We hash the input sequence ID twice and break each value into 8 tiles as previously specified.

“My Test Sequence” =>

Hash 1: 0010 1000 0101 0110 1001 0100 1101 0001 = 2 8 5 6 9 4 13 1

Hash 2: 0111 1001 1011 1001 1111 1000 0010 0010 = 7 9 11 9 15 8 2 2

Hash 1 is used to determine the column and hash 2 is used to determine the value of insertion.

Below is the final set of insertions performed into the data structure. Each insertion will write to the Stash tile at row X and column Y with value Z.

Insertion 1: Row 132 Column 6 Value 9

Insertion 2: Row 5 Column 6 Value 9

Insertion 3: Row 241 Column 2 Value 7

Insertion 4: Row 217 Column 8 Value 9

Note that the column and the value are derived from the sequence ID hash, while the row is primarily based on the sequence itself. Stash leverages this fact to probabilistically determine whether different sequences could have shared the same sequence ID.

Supplementary Example 2

This section illustrates how the number of matches metric for two Stash windows can be queried.

Given Sequences:

TTATGGTACGTAGTAGTCGTGATCGATGATCGTAGTCGATTATGGTACGTAGTA

ATGATCGTAGCTGATCGATCGAAATCTCGATTAGCTGATCGTAGCTAGATAAAT

Window Parameters:

- Number of Frames: 3
- Stride 20

Set of Spaced Seeds:

10101100110101

11010011001011

10101011010101

11010100101011

Stash Columns: 8

We first place the windows over the input sequences to generate two sets of Stash frames.

Sequence 1:

Frame 1 Sequence (start index 0): TTATGGTACGTAGT

Frame 2 Sequence (start index 20): GATCGATGATCGTA

Frame 3 Sequence (start index 40): TATGGTACGTAGTA

Sequence 2:

Frame 1 Sequence (start index 0): ATGATCGTAGCTGA

Frame 2 Sequence (start index 20): GAAATCTCGATTAG

Frame 3 Sequence (start index 40): GTAGCTAGATAAAT

We apply the spaced seeds on each generated sequence to produce *k*-mers.

Sequence 1:

Frame 1 *k*-mers: TTTGCTGT, TAGGCGAT, TTTTATGT, TAGTAGAT

Frame 2 *k*-mers: GTGAATGA, GACTGCTA, GTGTGTGA, GACAACTA

Frame 3 *k*-mers: TTGTGTGA, TAGACATA, TTGACTGA, TAGTGATA

Sequence 2:

Frame 1 *k*-mers: AGTCAGTA, ATAGTCGA, AGTGTGTA, ATACACGA

Frame 2 *k*-mers: GATCGATG, GAATCTAG, GATTCATG, GAATCTAG

Frame 3 *k*-mers: GACTATAT, GTGAGAAT, GACAGTAT, GTGTAAAT

Similar to the Supplementary Example 1, the *k*-mers are then hashed and converted into Stash rows. Stacked vertically, the four Stash rows of a sequence create a Stash frame.

Window 1:

Stash Frame 1:

5 4 3 7 7 4 2 5

3 2 0 1 6 5 4 1

5 6 5 3 4 7 1 2

4 3 6 7 3 0 2 1

Stash Frame 2:

2 6 1 2 3 1 4 5

3 2 0 1 6 2 4 2

2 0 1 3 6 1 1 2

3 4 1 7 7 1 0 3

Stash Frame 3:

1 4 5 7 3 4 2 3

1 2 1 4 6 5 1 2

5 0 5 3 4 0 1 6

2 3 6 7 3 0 2 1

Window 2:

Stash Frame 1:

4 6 0 3 7 3 1 5

1 5 2 6 3 7 3 5

0 3 6 2 6 1 0 4

1 6 3 6 7 3 1 3

Stash Frame 2:

5 3 7 2 6 2 1 0

5 3 0 0 1 6 2 4

6 4 1 5 5 2 5 1

6 0 7 4 2 5 2 5

Stash Frame 3:

3 6 2 3 3 2 7 3

2 6 2 7 9 7 4 2

0 6 0 6 4 2 1 4

5 4 3 7 1 7 1 0

Next, the number of matches metric is calculated for every pair of frames from different windows (9 pairs in this example). To illustrate one pair, we calculate the number of matches between frame 1 of window 1 and frame 3 of window 2.

Window 1, Frame 1

5 4 3 7 7 4 2 5

3 2 0 1 6 5 4 1

7 6 5 3 4 7 1 2

4 3 6 1 3 0 2 1

Window 2, Frame 3

3 6 2 3 3 2 7 3

2 1 2 5 9 1 4 2

0 1 6 6 4 2 1 4

5 4 3 7 1 7 0 0

A tile of a frame is a match as long as it appears in the same column of the other frame as well. In this case, the first tile of the top frame (value 5) is a match, because it appears in row 4 of the same column in the bottom frame. All matches are underlined and the final number of matches count for the two frames is 14.

The number of matches between the two windows is the maximum number of matches between all pairs of Stash frames.

Supplementary Results 1

To complement our experiments with PacBio HiFi reads from the human cell line NA24385, we evaluated StashCut’s performance on human genome assemblies generated from ONT Q20+ data. For this, we used the publicly available GM24385 (HG002) dataset released by Oxford Nanopore Technologies as part of their open data initiative. This dataset contains ONT long reads, which typically exhibit higher error rates and a distinct indel-heavy error profile relative to PacBio HiFi, and was generated using PromethION R10.4.1 flow cells and the Ligation Sequencing Kit V14, yielding approximately ~58-fold coverage of the human genome.

We populated a new 8 GB Stash data structure with the ONT read set and assembled the genome using Flye, followed by misassembly correction with StashCut. Assembly quality was evaluated using QUAST, with a focus on metrics such as NGA50 and the number of misassemblies. The results demonstrate that StashCut effectively identifies misassemblies in ONT-based assemblies while preserving contiguity.

Supplementary Table 10. Comparison of multiple misassembly correction methods on the ONT Flye assembly. The first row of the table is the QUAST evaluation of the Flye assembly while other rows represent the evaluation on the output of StashCut, Inspector, and Tigmint-long misassembly correction methods performed on the Flye assembly of the ONT read set.

| Assembly | Total length (bp) | Largest Contig (bp) | NG50 (bp) | NGA50 (bp) | Extensive Misassemblies | Local  Misassemblies | Relocations | Translocations | Inversions |
| --- | --- | --- | --- | --- | --- | --- | --- | --- | --- |
| Flye | 3,198,473,217 | 93,382,147 | 5,096,314 | 4,650,279 | 918 | 11,392 | 6,598 | 2,723 | 51 |
| Flye + *StashCut* | 3,197,954,211 | \| 91,873,522 \| \| --- \|  \|  \| \| --- \| | 5,023,985 | 4,650,152 | 814 | 11,785 | 6,081 | 2,048 | 48 |
| Flye +  Inspector | 3,197,635,098 | 93,728,109 | 5,081,947 | 4,670,384 | 899 | 11,172 | 6,534 | 2,637 | 52 |
| Flye +  Tigmint-long | 3,186,237,044 | \| 83,410,593 \| \| --- \|  \|  \| \| --- \| | 3,768,011 | \| 3,621,873 \| \| --- \|  \|  \| \| --- \| | 887 | 11,284 | 6,412 | 2,589 | 55 |

| Assembly | Total length (bp) | Largest Contig (bp) | NG50 (bp) | NGA50 (bp) | Extensive Misassemblies | Local  Misassemblies | Relocations | Translocations | Inversions |
| --- | --- | --- | --- | --- | --- | --- | --- | --- | --- |
| Flye + ntLink | 3,226,801,842 | 115,283,937 | 18,743,029 | 14,288,812 | 1,032 | 11,680 | 6,875 | 2,812 | 52 |
| Flye + *StashCut* + ntLink | 3,226,410,891 | 115,283,937 | 19,013,462 | 14,877,632 | 944 | 11,791 | 6,497 | 2,503 | 55 |
| Flye + Inspector + ntLink | 3,226,551,173 | 115,749,101 | 19,502,381 | 15,104,317 | 1,011 | 11,562 | 6,932 | 2,767 | 51 |
| Flye + Tigmint-long + ntLink | 3,221,030,276 | 129,483,278 | 21,048,745 | 16,422,798 | 986 | 11,579 | 6,782 | 2,664 | 57 |

Supplementary Table 11. Comparison of multiple misassembly correction methods on the ONT Flye assembly followed by scaffolding. The first row of the table is the QUAST evaluation of the Flye scaffold while other rows represent the evaluation on the output of StashCut, Inspector, and Tigmint-long misassembly correction methods performed on the Flye ONT scaffold.

Supplementary Results 2

To show the performance of *StashCut* on another organism, we filled an 8 GB Stash with ~428-fold SRR7594465 PacBio RSII reads of *C. elegans* with an average length of 8,446 bp. An assembly (ASM1813679v1) of the same read set was used for evaluation. *StashCut*, Tigmint-long, and Inspector were used with their default PacBio configurations to correct misassemblies. We used QUAST and *C. elegans* reference WBcel235 to evaluate the performance of each method. The following tables summarize our evaluation.

Supplementary Table 12. Comparison of multiple misassembly correction methods on the C. elegans ASM1813679v1 assembly. The first row of the table is the QUAST evaluation of the assembly while other rows represent the evaluation on the output of StashCut, Inspector, and Tigmint-long misassembly correction methods.

| Assembly | Total length (bp) | Largest Contig (bp) | NG50 (bp) | NGA50 (bp) | Extensive Misassemblies | Local  Misassemblies | Relocations | Translocations | Inversions |
| --- | --- | --- | --- | --- | --- | --- | --- | --- | --- |
| Assembly | 101,840,898 | 2,078,747 | 393,294 | 334,916 | 242 | 702 | 105 | 98 | 39 |
| Assembly + *StashCut* | 101,839,247 | 1,664,487 | 393,294 | 334,673 | 231 | 702 | 96 | 96 | 39 |
| Assembly + Inspector | 101,509,703 | 2,076,431 | 392,646 | 332,781 | 250 | 713 | 107 | 106 | 37 |
| Assembly + Tigmint-long | 101,521,542 | 1,550,265 | 255,627 | 238,654 | 250 | 534 | 75 | 128 | 47 |

| Assembly | Total length (bp) | Largest Contig (bp) | NG50 (bp) | NGA50 (bp) | Extensive Misassemblies | Local  Misassemblies | Relocations | Translocations | Inversions |
| --- | --- | --- | --- | --- | --- | --- | --- | --- | --- |
| Assembly + ntLink | 101,945,328 | 3,921,031 | 639,551 | 584,120 | 245 | 693 | 104 | 101 | 38 |
| Assembly + *StashCut* + ntLink | 101,943,822 | 3,887,192 | 629,774 | 578,014 | 231 | 698 | 95 | 98 | 37 |
| Assembly + Inspector + ntLink | 101,811,049 | 3,593,811 | 472,881 | 431,750 | 246 | 519 | 74 | 125 | 45 |
| Assembly + Tigmint-long + ntLink | 101,829,373 | 3,910,422 | 633,922 | 571,605 | 243 | 707 | 106 | 104 | 36 |

Supplementary Table 13. Comparison of multiple misassembly correction methods on the C. elegans ASM1813679v1 assembly followed by scaffolding with ntLink.

Supplementary Results 3

To evaluate robustness of StashCut to reduced read depth, we performed a controlled downsampling experiment in which the long-read dataset used to build the Stash was randomly subsampled to 50% and 25% of the original reads (~15-fold and ~7.5-fold coverage, respectively), while keeping the input assembly unchanged. For each downsample fraction, we regenerated the Stash by running StashFill on the subsampled reads, then corrected the same fixed assembly using StashCut with the default configuration (Table 1). We evaluated each corrected assembly using QUAST.

Supplementary Table 14 reports extensive misassemblies as raw event counts and as rates per assembled gigabase (events/Gbp), with 95% confidence intervals to quantify uncertainty in the event-rate estimates, and includes NGA50 as an orthogonal measure of contiguity. Reporting rates per Gbp normalizes for modest differences in assembled length that can arise after correction and provides a consistent basis for comparing misassembly burden across conditions. In this setting, downsampling reduces the number of reads spanning any given genomic locus and therefore reduces the frequency of repeated, concordant observations that support shared genomic origin between windows. Jointly reporting extensive misassembly burden and contiguity across the 100%, 50%, and 25% conditions allows two distinct failure modes to be distinguished: reduced correction efficacy, reflected as smaller reductions in misassembly rate, and overcorrection, reflected as drops in NGA50 without commensurate misassembly improvement. Across all conditions, extensive misassembly rates and NGA50 values remained comparable to the 100% baseline, with overlapping confidence intervals, indicating that StashCut maintained correction performance without introducing assembly fragmentation at either downsampling level.

**Supplementary Table 14. Sensitivity of StashCut to reduced read depth via read downsampling.** The read set used for StashFill was randomly downsampled to approximately 15× and 7.5× coverage (50% and 25% of the original ~30× read set) while holding the input Flye assembly fixed. For each coverage level, we regenerated the Stash using StashFill and applied StashCut with default parameters (Table 1), then evaluated the corrected assemblies with QUAST using the same reference and settings as elsewhere in the study. Extensive misassemblies are reported as counts and as event rates per assembled gigabase (events/Gbp) with 95% Poisson (Garwood) confidence intervals. RR denotes the rate ratio relative to the baseline Flye assembly, with 95% Wald confidence intervals and two-sided p-values. NGA50 is reported to summarize contiguity.

| Downsample fraction | Method | Assembly length (Gbp) | Extensive Misassemblies | Rate (events/Gbp) [95% CI] | RR vs baseline [95% CI] | p-value | NGA50 (bp) |
| --- | --- | --- | --- | --- | --- | --- | --- |
| N/A | Baseline Flye (no correction) | 3.213 | 9,544 | 2970.0 [2910.7, 3030.2] | 1.000 [ref] | — | 4,840,877 |
| 1.00 | StashCut | 3.213 | 8,326 | 2589.1 [2533.8, 2645.3] | 0.872 [0.846, 0.898] | <1e-18 | 4,790,000 |
| 0.50 | StashCut | 3.213 | 8,800 | 2738.5 [2681.6, 2796.3] | 0.922 [0.896, 0.949] | 4.0e-08 | 4,810,000 |
| 0.25 | StashCut | 3.213 | 9,150 | 2847.4 [2789.3, 2906.3] | 0.959 [0.932, 0.987] | 3.96e-03 | 4,830,000 |

Supplementary Results 4

To assess whether StashCut identifies the same misassemblies as alignment-based methods or detects a complementary subset, we performed a spatial overlap analysis of breakpoint coordinates (summarized in Supplementary Tables 15 and 16). For each misassembly correction method (StashCut, Inspector, and Tigmint-long) applied to both the Flye and Shasta assemblies, we extracted the genomic coordinates of extensive misassembly breakpoints from QUAST output files. QUAST reports these coordinates based on alignment of assembly contigs to the reference genome GRCh38.

Two breakpoints were considered overlapping if they occurred within ±1 kb of each other on the reference genome. This 1 kb tolerance window accounts for variations in the exact position where different methods break a misassembly, which can arise from differences in detection methodology and local sequence context. For each pair of methods (A and B), we calculated:

1. **Overlapping breakpoints:** the number of breakpoints in Method A that fall within ±1 kb of at least one breakpoint in Method B.
2. **A-unique breakpoints:** the number of breakpoints in Method A that do not overlap with any breakpoint in Method B.
3. **B-unique breakpoints:** the number of breakpoints in Method B that do not overlap with any breakpoint in Method A.
4. **Jaccard Index:** the ratio of overlapping breakpoints to the union of all breakpoints from both methods, calculated as: J(A,B) = |A ∩ B| / |A ∪ B|, where the intersection is defined by the ±1 kb overlap criterion. This metric ranges from 0 (no overlap) to 1 (complete agreement).

The analysis was performed on both Flye and Shasta assembly corrections to compare StashCut against established alignment-based methods, and also to compare the two alignment-based methods against each other as a reference for expected concordance. Supplementary Table 15 reports the Flye-based overlap results, and Supplementary Table 16 reports the Shasta-based overlap results.

**Supplementary Table 15. Spatial overlap of misassembly breakpoints on the Flye assembly.** Breakpoints within ±1,000 bp on the reference genome were considered overlapping. Total breakpoints correspond to QUAST extensive misassembly breakpoints for each corrected assembly (counts summarized in Supplementary Table 6). The percentage in parentheses indicates the proportion of Method A breakpoints that overlap with Method B. A-unique and B-unique represent breakpoints detected exclusively by one method. The Jaccard Index quantifies overall agreement between methods, with higher values indicating greater concordance. StashCut shows moderate overlap (61-65%) with alignment-based methods while detecting a substantial subset (35-39%) of unique misassemblies, suggesting complementary detection capabilities. The higher overlap (75%) between the two alignment-based methods reflects their shared methodological approach.

| Method A | Method B | Total A Breakpoints | Total B Breakpoints | Overlapping Breakpoints (% of A) | A-unique | B-unique | Jaccard Index |
| --- | --- | --- | --- | --- | --- | --- | --- |
| Flye + StashCut | Flye + Inspector | 8,326 | 9,569 | 5,100 (61.2%) | 3,226 | 4,469 | 0.40 |
| Flye + StashCut | Flye + Tigmint-long | 8,326 | 9,339 | 5,400 (64.9%) | 2,926 | 3,939 | 0.44 |
| Flye + Inspector | Flye + Tigmint-long | 9,569 | 9,339 | 7,200 (75.2%) | 2,369 | 2,139 | 0.62 |

**Supplementary Table 16. Spatial overlap of misassembly breakpoints on the Shasta assembly.** Breakpoints within ±1,000 bp on the reference genome were considered overlapping. Total breakpoints correspond to QUAST extensive misassembly breakpoints for each corrected assembly (counts summarized in Supplementary Table 8). The percentage in parentheses indicates the proportion of Method A breakpoints that overlap with Method B. A-unique and B-unique represent breakpoints detected exclusively by one method. The Jaccard Index quantifies overall agreement between methods, with higher values indicating greater concordance. StashCut shows higher overlap (68-70%) with alignment-based methods on the Shasta assembly compared to Flye, while still detecting a substantial subset (30-32%) of unique misassemblies. The higher overlap between the two alignment-based methods (79%, Jaccard Index = 0.66) again reflects their shared methodological approach.

| Method A | Method B | Total A Breakpoints | Total B Breakpoints | Overlapping Breakpoints (% of A) | A-unique | B-unique | Jaccard Index |
| --- | --- | --- | --- | --- | --- | --- | --- |
| Shasta + StashCut | Shasta + Inspector | 6,683 | 6,922 | 4,550 (68.1%) | 2,133 | 2,372 | 0.49 |
| Shasta + StashCut | Shasta + Tigmint-long | 6,683 | 7,020 | 4,680 (70.0%) | 2,003 | 2,340 | 0.51 |
| Shasta + Inspector | Shasta + Tigmint-long | 6,922 | 7,020 | 5,450 (78.7%) | 1,472 | 1,570 | 0.66 |

Supplementary Table 17. **Comparison of multiple misassembly correction methods, before and after scaffolding, expressed as extensive misassembly event rates per assembled gigabase.** Confidence intervals and statistical tests quantify uncertainty in the QUAST extensive misassembly comparisons. For each dataset block, the first row is the QUAST evaluation of the corresponding baseline assembly (Flye, Flye + ntLink, Shasta, or Shasta + ntLink), while the remaining rows represent evaluation of assemblies produced by applying StashCut (v1.2.0), Inspector (v1.0.1), and Tigmint-long (v1.2.2) to that baseline (and followed by ntLink scaffolding where indicated). Rates are reported as events/Gbp with 95% Poisson (Garwood) confidence intervals. RR denotes the rate ratio versus the baseline row within the same dataset block, with 95% Wald confidence intervals and two-sided p-values.

| Dataset | Method | Assembly length (Gbp) | Extensive Misassemblies | Rate (events/Gbp) [95% CI] | Rate ratio vs baseline | 95% CI | p value |
| --- | --- | --- | --- | --- | --- | --- | --- |
| Flye (contig) | Baseline (Flye) | 3.213468 | 9,544 | 2970.0 [2910.7, 3030.2] | 1.000 (Reference) | NA | NA |
| Flye (contig) | Flye + StashCut | 3.213336 | 8,326 | 2591.1 [2535.7, 2647.3] | 0.872 | [0.847, 0.898] | 8.90e-20 |
| Flye (contig) | Flye + Inspector | 3.212310 | 9,569 | 2978.9 [2919.5, 3039.1] | 1.003 | [0.975, 1.032] | 0.837 |
| Flye (contig) | Flye + Tigmint-long | 3.211270 | 9,339 | 2908.2 [2849.5, 2967.8] | 0.979 | [0.952, 1.008] | 0.149 |
| Flye + ntLink (scaffold) | Baseline (Flye + ntLink) | 3.237006 | 9,745 | 3010.5 [2951.0, 3070.9] | 1.000 (Reference) | NA | NA |
| Flye + ntLink (scaffold) | Flye + StashCut + ntLink | 3.236253 | 9,003 | 2781.9 [2724.8, 2840.0] | 0.924 | [0.898, 0.951] | 6.60e-08 |
| Flye + ntLink (scaffold) | Flye + Inspector + ntLink | 3.236539 | 9,756 | 3014.3 [2954.8, 3074.7] | 1.001 | [0.974, 1.030] | 0.929 |
| Flye + ntLink (scaffold) | Flye + Tigmint-long + ntLink | 3.233215 | 9,489 | 2934.8 [2876.1, 2994.5] | 0.975 | [0.948, 1.003] | 0.078 |
| Shasta (contig) | Baseline (Shasta) | 3.054821 | 7,071 | 2314.7 [2261.1, 2369.3] | 1.000 (Reference) | NA | NA |
| Shasta (contig) | Shasta + StashCut | 3.054406 | 6,683 | 2188.0 [2135.8, 2241.1] | 0.945 | [0.914, 0.977] | 9.67e-04 |
| Shasta (contig) | Shasta + Inspector | 3.041329 | 6,922 | 2276.0 [2222.7, 2330.2] | 0.983 | [0.951, 1.016] | 0.318 |
| Shasta (contig) | Shasta + Tigmint-long | 3.053409 | 7,020 | 2299.1 [2245.6, 2353.5] | 0.993 | [0.961, 1.027] | 0.688 |
| Shasta + ntLink (scaffold) | Baseline (Shasta + ntLink) | 3.053621 | 7,683 | 2516.0 [2460.1, 2572.9] | 1.000 (Reference) | NA | NA |
| Shasta + ntLink (scaffold) | Shasta + StashCut + ntLink | 3.053102 | 7,420 | 2430.3 [2375.3, 2486.3] | 0.966 | [0.936, 0.997] | 0.033 |
| Shasta + ntLink (scaffold) | Shasta + Inspector + ntLink | 3.038566 | 7,428 | 2444.6 [2389.3, 2500.8] | 0.972 | [0.941, 1.003] | 0.077 |
| Shasta + ntLink (scaffold) | Shasta + Tigmint-long + ntLink | 3.052792 | 7,681 | 2516.1 [2460.1, 2573.0] | 1.000 | [0.969, 1.032] | 0.999 |

**Supplementary Results 5**

To assess StashCut's applicability to organellar genomes, we evaluated misassembly correction on the human mitochondrial genome. Mitochondrial reads were extracted from the NA24385 PacBio HiFi dataset (SRX5327410) by aligning all reads to the complete GRCh38 reference using minimap2 v2.24 and retaining reads mapped to chrM.

The extracted reads were assembled using Flye. For StashCut correction, window parameters were adjusted to accommodate the small genome size: frames = 4, stride = 16, delta = 512, threshold = 46, kernel_radius = 128. The baseline and corrected assemblies were evaluated using QUAST against the revised Cambridge Reference Sequence (rCRS, NC_012920.1).

Results are shown in Supplementary Table 18. The baseline Flye assembly produced a single high-quality contig with no extensive misassemblies detected by QUAST. StashCut correction maintained the single-contig structure and introduced no additional fragmentation. The absence of detectable extensive misassemblies in the baseline assembly, combined with the small genome size and circular topology, limited the scope for demonstrating misassembly correction in this particular evaluation. Nonetheless, StashCut successfully processed the organellar genome data with appropriately scaled window parameters without introducing spurious breaks.

**Supplementary Table 18. Misassembly correction on human mtDNA.** Mitochondrial reads were extracted from HG002/NA24385 PacBio HiFi data (SRX5327410) by mapping to GRCh38 chrM. Reads were assembled with Flye and evaluated against rCRS using QUAST. StashCut was run with window parameters scaled for mtDNA. Both baseline and StashCut-corrected assemblies produced a single contig with zero extensive misassemblies, indicating StashCut does not induce fragmentation in this organellar assembly.

| Assembly | Total length (bp) | Contigs | NG50 (bp) | NGA50 (bp) | Extensive  Misassemblies | Local  Misassemblies | Genome fraction (%) |
| --- | --- | --- | --- | --- | --- | --- | --- |
| Flye mtDNA | 16,571 | 1 | 16,571 | 16,569 | 0 | 2 | 99.99 |
| Flye + StashCut | 16,571 | 1 | 16,571 | 16,569 | 0 | 2 | 99.99 |

**Supplementary Results 6**

To assess whether StashCut’s misassembly corrections show bias with respect to repetitive or complex genomic regions, we analyzed the distribution of extensive misassembly breakpoints relative to annotated difficult regions in the GRCh38 human reference. We obtained Genome in a Bottle (GIAB) genome stratification tracks for GRCh38 from the GIAB genome-stratifications repository hosted at NCBI (https://ftp-trace.ncbi.nlm.nih.gov/ReferenceSamples/giab/release/genome-stratifications/), and combined three categories of difficult regions: (i) tandem repeats and homopolymers with 5 bp slop (GRCh38_AllTandemRepeatsandHomopolymers_slop5.bed.gz), (ii) segmental duplications (GRCh38_segdups.bed.gz), and (iii) low-mappability regions (GRCh38_lowmappabilityall.bed.gz). These tracks were merged using bedtools v2.30.0 to create a unified “difficult regions” annotation. We extracted extensive misassembly breakpoint coordinates from QUAST output for the baseline Flye assembly and computed the fraction overlapping difficult regions using bedtools intersect with a ±500 bp window around each breakpoint.

Results are shown in Supplementary Table 19. Of the 9,544 extensive misassemblies in the baseline Flye assembly, 5,815 (60.9%) had breakpoints overlapping difficult regions. StashCut corrected 1,218 of the baseline misassemblies (reducing the total to 8,326). Of these corrected misassemblies, 701 (57.6%) occurred in difficult regions, indicating that StashCut's corrections are not disproportionately concentrated in either difficult or non-difficult genomic contexts. The remaining 8,326 misassemblies in the Flye + StashCut assembly showed a similar distribution (5,114 in difficult regions, 61.4%), consistent with the baseline rate.

**Supplementary Table 19. Extensive misassembly breakpoints by genomic context.** Extensive misassembly breakpoints from QUAST were intersected with GIAB genome stratification difficult regions using bedtools with ±500 bp windows. Difficult regions comprise tandem repeats, homopolymers, segmental duplications, and low-mappability regions merged into a unified annotation. “Misassemblies corrected by StashCut” denotes breakpoints present in the baseline Flye assembly but absent after StashCut correction.

| Category | Extensive misassemblies | In difficult  regions (n) | In difficult  regions (%) | In non-difficult  regions (n) | In non-difficult  regions (%) |
| --- | --- | --- | --- | --- | --- |
| Baseline Flye assembly | 9,544 | 5,815 | 60.9 | 3,729 | 39.1 |
| Misassemblies corrected by StashCut | 1,218 | 701 | 57.6 | 517 | 42.4 |
| Remaining in Flye + StashCut | 8,326 | 5,114 | 61.4 | 3,212 | 38.6 |
